# Exploring the Anticancer Effects of Brominated Plastoquinone Analogs with Promising Cytotoxic Activity in MCF-7 Breast Cancer Cells via Cell Cycle Arrest and Oxidative Stress Induction

**DOI:** 10.3390/ph15070777

**Published:** 2022-06-22

**Authors:** Ayse Tarbin Jannuzzi, Ayse Mine Yilmaz Goler, Nilüfer Bayrak, Mahmut Yıldız, Hatice Yıldırım, Betul Karademir Yilmaz, Deepak Shilkar, Raghusrinivasan Jayaprakash Venkatesan, Venkatesan Jayaprakash, Amaç Fatih TuYuN

**Affiliations:** 1 Department of Pharmaceutical Toxicology, Faculty of Pharmacy, Istanbul University, Beyazit, Istanbul 34116, Turkey; tarbin.cevik@istanbul.edu.tr; 2 Department of Biochemistry, School of Medicine, Marmara University, Istanbul 34854, Turkey; ayse.mine@marmara.edu.tr (A.M.Y.G.); betulkarademir@marmara.edu.tr (B.K.Y.); 3 Genetic and Metabolic Diseases Research and Investigation Center, Marmara University, Istanbul 34854, Turkey; 4 Department of Chemistry, Faculty of Engineering, Istanbul University-Cerrahpasa, Avcılar, Istanbul 34320, Turkey; nbayrak@istanbul.edu.tr (N.B.); hyildirim@iuc.edu.tr (H.Y.); 5 Chemistry Department, Gebze Technical University, Gebze, Kocaeli 41400, Turkey; yildizm@gtu.edu.tr; 6 Department of Pharmaceutical Sciences & Technology, Birla Institute of Technology, Mesra, Ranchi 835215, India; deepakshilkar@live.com (D.S.); venkatesanj@bitmesra.ac.in (V.J.); 7 Department of Industrial and Systems Engg, Indian Institute of Technology, Kharagpur 721302, India; rover.rags@gmail.com; 8 Department of Chemistry, Faculty of Science, Istanbul University, Fatih, Istanbul 34126, Turkey

**Keywords:** quinone, plastoquinones, antiproliferative activity, cytotoxicity, cell cycle, oxidative stress, ADME

## Abstract

Plastoquinone analogs are privileged structures among the known antiproliferative natural product-based compound families. Exploiting one of these analogs as a lead structure, we report the investigation of the brominated PQ analogs (BrPQ) in collaboration with the National Cancer Institute of Bethesda within the Developmental Therapeutics Program (DTP). These analogs exhibited growth inhibition in the micromolar range across leukemia, non-small cell lung cancer (EKVX, HOP-92, and NCI-H522), colon cancer (HCT-116, HOP-92), melanoma (LOX IMVI), and ovarian cancer (OVCAR-4) cell lines. One brominated PQ analog (**BrPQ5**) was selected for a full panel five-dose in vitro assay by the NCI’s Development Therapeutic Program (DTP) division to determine GI_50_, TGI, and LC_50_ parameters. The brominated PQ analog (**BrPQ5**) displayed remarkable activity against most tested cell lines, with GI_50_ values ranging from 1.55 to 4.41 µM. The designed molecules (BrPQ analogs) obeyed drug-likeness rules, displayed a favorable predictive Absorption, Distribution, Metabolism, and Excretion (ADME) profile, and an in silico simulation predicted a possible **BrPQ5** interaction with proteasome catalytic subunits. Furthermore, the in vitro cytotoxic activity of **BrPQ5** was assessed, and IC_50_ values for U-251 glioma, MCF-7 and MDA-MB-231 breast cancers, DU145 prostate cancer, HCT-116 colon cancer, and VHF93 fibroblast cell lines were evaluated using an MTT assay. MCF-7 was the most affected cell line, and the effects of **BrPQ5** on cell proliferation, cell cycle, oxidative stress, apoptosis/necrosis induction, and proteasome activity were further investigated in MCF-7 cells. The in vitro assay results showed that **BrPQ5** caused cytotoxicity in MCF-7 breast cancer cells via cell cycle arrest and oxidative stress induction. However, **BrPQ5** did not inhibit the catalytic activity of the proteasome. These results provide valuable insights for further discovery of novel antiproliferative agents.

## 1. Introduction

Breast cancer is a malignant tumor affecting areas in or around the breast tissue [1]. There are different types of breast cancer, depending on which cells in the breast become cancerous. Lobules, ducts, and connective tissue are three key parts of the breast [2]. Breast cancer affects the ducts of the glandular tissue of the breast in 85% of the cases or the lining cells of the lobules in 15% of the cases [3]. Cancerous growth initially occurs only in the duct or lobule, usually without causing any symptoms, and the potential to spread (metastasize) is minimal. Metastasis is responsible for the fatal outcomes associated with breast cancer [3]. The statistical data published by the International Agency for Research on Cancer (IARC) in December 2020 suggests that approximately 2.3 million women were diagnosed with breast cancer in 2020, and breast cancer caused approximately 685,000 deaths. Before World Cancer Day 2021, the WHO experts announced that the global cancer landscape had changed significantly and declared that breast cancer had surpassed lung cancer as the most frequently diagnosed type of cancer worldwide. Vowing to fight the disease, the WHO created a new global breast cancer initiative and has been holding necessary consultations [4]. The key treatments for breast cancer include surgery, chemotherapy, radiotherapy, hormonal therapy, bone-strengthening drugs, and molecular-targeted anticancer drugs [5,6]. Despite the availability of many anticancer drugs to treat breast cancer, the pursuit of developing novel anticancer drugs remains the primary focus of pharmaceutical companies and scientists, considering the demand for less toxic, safe, and effective drugs [7,8]. With the recent trend of synthesizing new compounds, often based on biologically active small molecules, organic synthesis aims to bridge the gap between cancer and new drugs that treat this disease.

Aminoquinones continue to attract significant attention as drug candidates because of their broad biological activity profiles and easy accessibility [9,10,11]. Many studies have been conducted on the biological activities of quinones, particularly focusing on the anticancer and antimicrobial properties of aminoquinones [12,13,14,15,16,17,18,19]. Recent developments in our group have contributed significantly to searching and identifying the lead molecules based on natural products, mainly focusing on the antiproliferative [20,21,22] and antimicrobial profiles [23,24] of quinones. The common characteristic within the lead structures is the presence of a primary (aryl) or secondary amine and 1,4-quinone moieties. The potential complementarity and diversity of these moieties offer a significant scope for synthesizing new molecules. Our previous studies have indicated that the halogenated plastoquinone (PQ) analogs (brominated or chlorinated) are more active than the nonhalogenated PQ analogs [23,25,26]. The chlorinated PQ analogs exhibited maximum sensitivity toward many cancer cell lines, particularly leukemia cell lines, than the nonhalogenated PQ analogs [27]. Assessing the anticancer profiles of previously obtained PQ analogs revealed that the different substitutent(s) at different positions in the arylamino moiety and chlorine atom in the quinone moiety were essential for the analogs’ activity, and their absence led to the loss of activity in most cases. Therefore, the arylamino moiety and bromine atom (instead of chlorine atom) were preserved in these analogs. Our design aimed at two different structural modifications on the PQ analogs. Based on these encouraging results, our research group focused on investigating the BrPQ analogs for their antiproliferative activity against versatile cancer cell lines. In search of new antiproliferative agents, and in continuation of our research focus on discovering new anti-infective small molecules [28], we investigated the antiproliferative profile of the BrPQ analogs with a variety of substituents in different positions on the aminophenyl moiety. In line with these observations, the present study focused on the anticancer effects of a selected molecule, **BrPQ5**. The cytotoxic activity of **BrPQ5** was evaluated against U-251 glioma, MCF-7 and MDA-MB-231 breast cancers, DU145 prostate cancer, and HCT-116 colon cancer cell lines. Furthermore, the IC_50_ values were calculated. These results were compared with VHF93 fibroblast cells to determine cancer selectivity. To better elucidate the mechanisms underlying the cytotoxic activity of **BrPQ5** against the most sensitive cell line, MCF-7, the cell proliferation was evaluated through its colony-forming ability. Moreover, we investigated the effect of **BrPQ5** on apoptosis induction and cell cycle profile in MCF7 cells. The role of apoptotic/necrotic cell death and cell cycle changes on the decrease in cell proliferation was investigated. An excessive accumulation of reactive oxygen species (ROS) in cells can lead to oxidative stress-induced cellular damage [29]. Thus, the intracellular ROS level was also measured. Molecular docking studies against proteasome catalytic sites were performed with **BrPQ5** as the ligand to study its binding mode, and proteasome activity was measured in MCF7 cells to confirm its inhibitory activity.

## 2. Results and Discussion

### 2.1. Design Strategy

**Figure 1:**
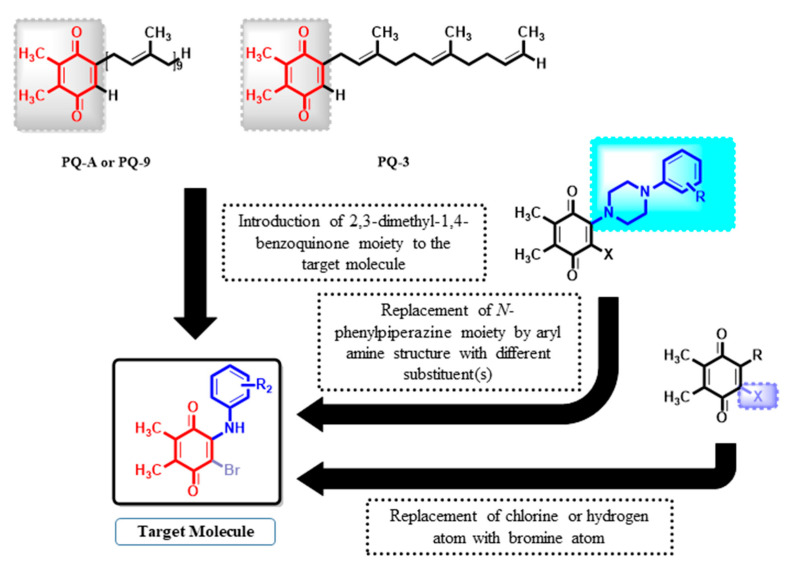
The rationale underlying the design of BrPQ analogs based on previous studies.

Drugs such as mitomycin C, mitoxantrone, and doxorubicin and some of the biologically active synthetic molecules contain 1,4-quinone moiety. Our strategy was inspired by a natural 2,3-dimethyl-1,4-benzoquinone structure with a side chain of nine isoprenyl groups named PQ-A or PQ-9 [30], as well as those containing shorter side chains such as PQ-3, having three isoprenyl side units as shown in Figure 1 [31]. In the past three years, we have published the syntheses and extensive exploration of a broad range of various PQ analogs to elucidate their antiproliferative and/or antibiotic profiles using 2,3-di-methylhydroquinone as a precursor [21,23,26,28,32,33]. Figure 1 illustrates the design concept of BrPQ analogs. Based on our previous studies, we have combined different active parts of plastoquinone analogs into a single target molecule. The studied PQ analogs within this study were published as antimicrobial agents by a repositioning strategy previously by our group [25,28].

### 2.2. Biological Activities

#### 2.2.1. Preliminary Screening of the In Vitro Antiproliferative Activity

**Figure 2:**
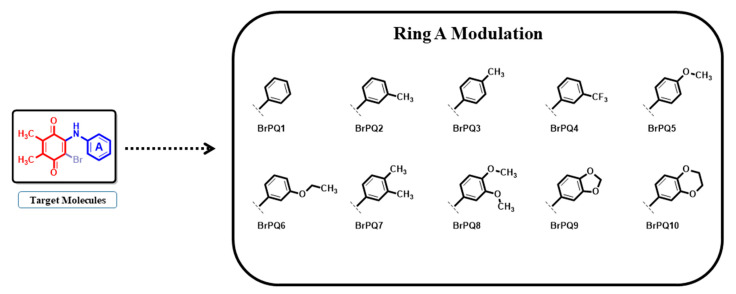
Structures of the BrPQ analogs (**BrPQ1–10**) explored in this study.

A preliminary antiproliferative in vitro assay (at a single dose of concentration, 10 µM) was performed under the Developmental Therapeutics Program (DTP) at the National Cancer Institute (NCI), Bethesda, to explore the cytotoxic activity of the BrPQ analogs against a panel of 60 human cancer cell lines, which included nine tumor subpanels, namely leukemia, lung, colon, CNS, melanoma, ovarian, renal, prostate, and breast cancer cell lines [34]. Before biological testing, the purity of the BrPQ analogs was analyzed using HPLC (Shimadzu/DGU-20A5 HPLC apparatus fitted with a 25 cm Chiralpac AD-H chiral column) with hexane/2-propanol [95:5] as the mobile phase at a flow rate of 1.0 mL/min. The purity of all analogs was ≥95%. Their chromatograms are provided as Supplementary Material (Figures S1–S10). The preclinical development program of NCI plays a vital role in finding and developing the lead molecules for the next stage in drug discovery worldwide. Out of all the presented analogs, herein, 10 BrPQ analogs, namely **BrPQ1** (NCI: D-825199/1), **BrPQ2** (NCI: D-827603/1), **BrPQ3** (NCI: D-827604/1), **BrPQ4** (NCI: D-827602/1), **BrPQ5** (NCI: D-825197/1), **BrPQ6** (NCI: D-825198/1), **BrPQ7** (NCI: D-827608/1), **BrPQ8** (NCI: D-827605/1), **BrPQ9** (NCI: D-827606/1), and **BrPQ10** (NCI: D-827607/1) were tested by the NCI for an in vitro disease-oriented human cell screening panel assay. The structures of the BrPQ analogs are shown in Figure 2. Among the ten BrPQ analogs, five BrPQ analogs (**BrPQ2**, **BrPQ5**, **BrPQ6**, **BrPQ9**, and **BrPQ10**) exhibited broad-spectrum potent inhibitory effects on some leukemia cell lines. Furthermore, three BrPQ analogs (**BrPQ6**, **BrPQ7**, and **BrPQ9**) showed strong antiproliferative activity against breast cancer cell lines. Additionally, **BrPQ5** was selected for a five-dose level screening for further in vitro evaluation. Details of the antiproliferative activity results at one-dose and five-dose analyses are provided separately under two subtitles.

#### 2.2.2. In Vitro Antiproliferative Activity at the One-Dose Assay

The observed one-dose results of each tested BrPQ analog were reported as a function of the growth percentage (GP) of treated cells. Their one-dose mean graphs and table are presented in the Supplementary Material as Figures S11–S20 and Table S1. All BrPQ analogs did not exert an acceptable inhibitory effect on some cancer cell lines, such as CNS cancer, melanoma (except the LOX IMVI cell line), ovarian cancer (except the OVCAR-4 cell line), renal cancer, and prostate cancer. Among all the BrPQ analogs, the BrPQ analogs with alkoxy substituent(s) in the amino phenyl moiety displayed maximum sensitivity toward several cancer cell lines, particularly CCRF-CEM, HL-60(TB), K-562, MOLT-4, and SR leukemia cell lines, as shown in Table S1. Looking specifically at those analogs suggests that they also displayed considerable antiproliferative activity against breast cancer cell lines. Prominent activity was also observed against breast cancer cell lines. Four BrPQ analogs (**BrPQ3**, **BrPQ4**, **BrPQ5,** and **BrPQ7**) displayed remarkable antiproliferative activity against the colon HCT-116 cell line, with a growth inhibition of 64.64, 79.99, 85.93, and 65.27%, respectively. **BrPQ3**, **BrPQ4,** and **BrPQ10** showed the best GI% values of 70.14, 93.36, and 79.67%, respectively, in the subpanel cell line of the melanoma cancer panel (LOX IMVI). Surprisingly, among the brominated PQ analogs, only one analog (**BrPQ8**) displayed maximum sensitivity against the ovarian OVCAR-4 cell line with a growth inhibition percentage of 85.94%. Moreover, two analogs (**BrPQ4** and **BrPQ7**) exhibited excellent to moderate antiproliferative activity in some subpanel cell lines of the non-small cell lung cancer panel (EKVX, HOP-92, and NCI-H522). **BrPQ7** was the most potent analog with above 90% GI% values for the colon cancer line HOP-92. The bromi-nated PQ analogs showed a significant antiproliferative effect profile against leukemia and breast cancer models. **BrPQ2** displayed maximum sensitivity toward K-562 and CCRF-CEM cancer cell lines of leukemia with 83.46 and 97.97% of GI%, respectively. It also showed promising activity against the T-47D (95.53%) breast cancer cell line. Three BrPQ analogs (**BrPQ6**, **BrPQ9,** and **BrPQ10**) showed a significant growth inhibition against K-562 with 99.57, 96.57, and 95.54%, respectively. The most potent BrPQ analog was the **BrPQ5** against the SR cell line of leukemia with 97.94% of GI%. **BrPQ6** exhibited the highest activity against CCRF-CEM (96.65%, GI%) and MOLT-4 (97.43%, GI%) cell lines of the leukemia panel. On the other hand, the most endowed BrPQ analog (**BrPQ7**) displayed potency against the MDA-MB-468 (98.74%, GI%) breast cancer cell line.

#### 2.2.3. In Vitro Antiproliferative Activity at Five-Dose Assay

**Figure 3:**
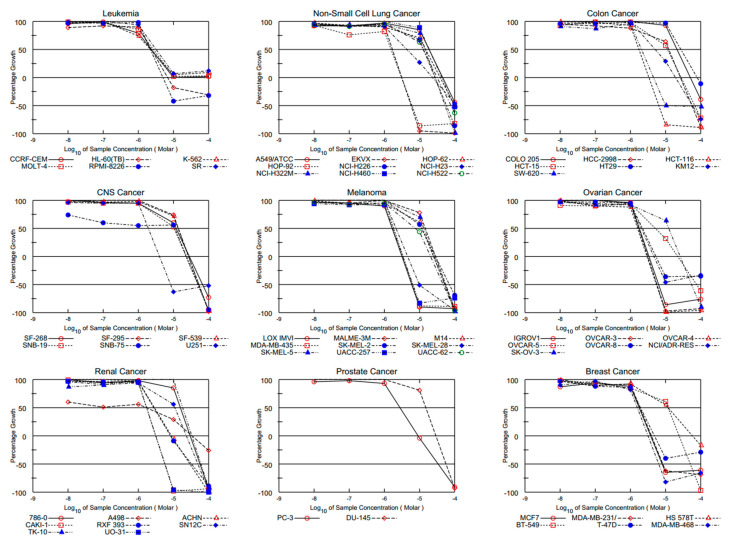
All five-dose-response curves of at NCI fixed protocol of the BrPQ analog (**BrPQ5**).

The brominated PQ analog (**BrPQ5**, NCI: D-825197/1) satisfied the pre-determined threshold inhibition criteria in a minimum number of cell lines and was advanced to the five-dose antiproliferative screening against the 60 cell panel at five-dose concentrations and 10-fold dilutions of five different concentrations (ranging from 0.01 µM to 100 µM). The five-dose mean graphs are presented in the Supplementary Material as Figures S21–S23. The generated dose–response curves enable the determination of three response parameters, namely GI_50_ (concentration at which 50% of growth inhibitory activity, µM), TGI (total growth inhibition, µM), and LC_50_ (concentration at which 50% of cancer cells are killed, µM), presented in Table 1 for each cell line on nine panels of human cancer cell lines [34,35]. The results obtained from the preliminary in vitro antiproliferative activity at five-dose concentration levels are shown in Table 1. The most sensitive cancer cell lines were all leukemia cell lines, EKVX, HOP-92, NCI-H23 (non-small cell lung cancer cell lines), HCT-116, SW-620 (colon cancer cell lines), LOX IMVI, MDA-MB-435, SK-MEL-28, UACC-257 (melanoma cell lines), IGROV1, OVCAR-3, OVCAR-4, OVCAR-8, NCI/ADR-RES (ovarian cancer cell lines), A498, ACHN, CAKI-1, RXF 393, UO-31 (renal cancer cell lines), PC-3 (prostate cancer cell line), and MCF7, MDA-MB-231/ATCC, T-47D, MDA-MB-468 (breast cancer cell lines), with GI_50_ values ranging from 1.55 to 4.41 µM. An excellent potency was seen for the selected BrPQ analog (**BrPQ5**) against the breast cancer cell lines MCF7, MDA-MB-231/ATCC, T-47D, and MDA-MB-468. Additionally, the analog **BrPQ5** showed high activity with GI_50_ values ranging from 2.21 to 3.21 µM. It also displayed a pronounced activity against other cancer cell lines from different tumor subpanels. Moreover, this analog possessed promising TGI values ranging from 3.16 to 4.81 µM against some breast cancer cell lines. Additionally, significant TGI values ranging from 5.00 to 10.00 µM were noticed with some cancer cell lines. Concerning the lethality (LC_50_ values), a few panel cancer cell lines showed values exceeding 100 µM. Finally, it displayed potent lethal action against some cancer cell lines (LC_50_ values from 5.00 to 20.00 µM). All the five-dose–response curves of the BrPQ analog (**BrPQ5**) against the panel of 60 human cancer cell lines are presented in Figure 3.

#### 2.2.4. in silico Study

High oral bioavailability is a key factor that is essential for introducing biologically active molecules into the therapeutic market. Oral bioavailability can be predicted by examining molecular features, i.e., low polar surface area or total hydrogen bonding, reduced molecular flexibility, and good intestinal absorption [36,37]. All molecular properties (Absorption, Distribution, Metabolism, and Excretion (ADME) parameters, pharmacokinetic profile with drug-likeness) and associated descriptive parameters of the BrPQ analogs (**BrPQ5**) were predicted using the free web tool SwissADME (http://www.swissadme.ch, accessed on 3 May 2022) developed by the Swiss Institute of Bioinformatics [38], and are reported in the Supplementary Material as Figure S24. The compound was found to have significantly favorable pharmacokinetic and physicochemical properties, accounting for its flexibility, lipophilicity, size, polarity, solubility, and unsaturation. The BrPQ analog (**BrPQ5**) displayed a good lipophilic character with a consensus Log *P*_o/w_ value of 2.75 and moderate solubility in water. The pink area in the bioavailability radar chart (in the Supplementary Material as Figure S24) represents the values most advantageous for oral bioavailability, i.e., flexibility (FLEX), lipophilicity (LIPO), size (SIZE), polarity (POLAR), and solubility (INSOLU), except for saturation (INSATU) [38,39]. Lipinski’s rule of five was used as a filter for drug-like properties by stating good membrane permeability of molecules with MW ≤ 500 (molecular weight), log P ≤ 5, N or O ≤ 10 (number of H-bond acceptor, HBA), NH or OH ≤ 5 (number of H-bond donor, HBD), n_Rot_ ≤ 10 (number of rotatable bonds), and TPSA < 140 Å^2^ (topological polar surface area, TBSA). The BrPQ analog (**BrPQ5**) carries all the required properties without violating Lipinski’s rule of five. As a biologically active molecule containing less than or equal to five hydrogen bond donors and less than 10 hydrogen bond acceptors, **BrPQ5** is likely to have rich absorption and cellular permeation.

The calculation method for SwissADME is an accurate predictive model (Brain Or IntestinaL EstimateD permeation, BOILED-Egg) developed for lead optimization by calculating the lipophilicity and polarity of small molecules [40]. The BrPQ analog (**BrPQ5**) showed high gastrointestinal (GI) absorption according to the white of the BOILED-Egg, illustrated in the Supplementary Material as Figure S25. The BrPQ analog (**BrPQ5**), as indicated by the BOILED-Egg graph, was predicted to passively permeate through the blood–brain barrier (BBB) according to the yolk portion of the BOILED-Egg chart. Additionally, the BrPQ analog (**BrPQ5**) was unsuitable as a substrate for P-glycoprotein (P-gp) according to the BOILED-Egg graph. The observed results from an in silico ADME study indicate the potential pharmacological use of the BrPQ analog (**BrPQ5**) that would be a suitable template for further studies to determine the clinical applications in specific leukemia types.

**Figure 4:**
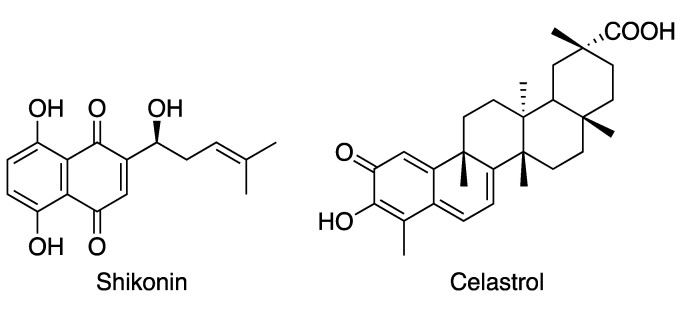
Proteasome inhibitors having a quinone pharmacophore.

The analysis of cell cycle and oxidative stress in MCF-7 breast cancer cell lines after administering **BrPQ5** suggested that it blocks the cell cycle at the G0/G1 phase with the induction of the S phase and an increase in oxidative stress. Proteasome inhibitors show a few characteristic features. Additionally, **BrPQ5** shares structural similarity with known proteasome inhibitors of natural origin, shikonin and celastrol, as they also have a quinone pharmacophore (Figure 4). This prompted us to perform a simulation study for **BrPQ5** with human 20S proteasome beta-1 (caspase-like), beta-2 (trypsin-like), and beta-5 (chymotrypsin-like) subunits. The X-ray crystal structure for human 20S proteasome in complex with the proteasome inhibitor carfilzomib (PDB: 4R67) was used for the simulation. **BrPQ5** was found to interact well with all the three subunits, and the interactions are discussed below.

**Figure 5:**
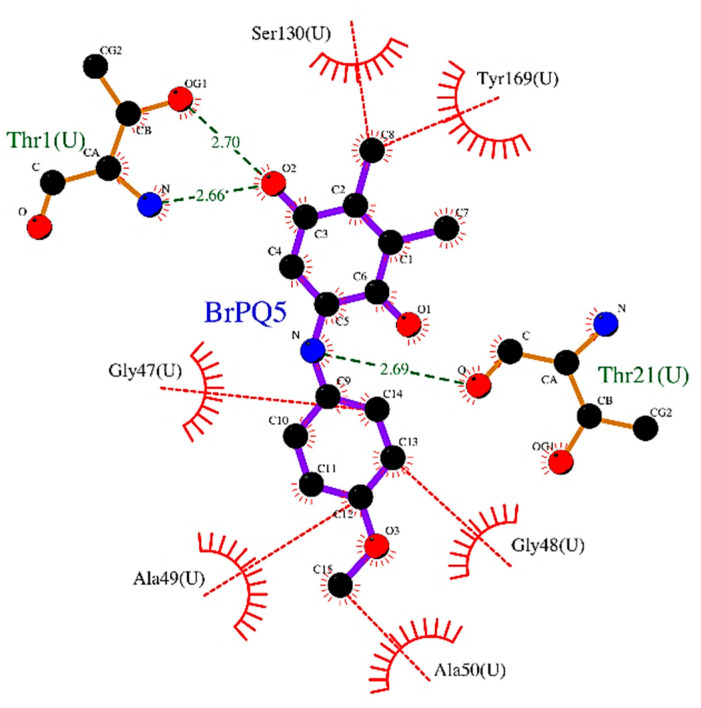
2D-plot of **BrPQ5** in complex with beta5 subunit of the human 20S proteasome. Atoms are colored by atom type. Bonds of ligand are shown with violet lines. Bonds of amino acids are shown as orange lines. H-bonds are shown as green dashed lines. Hydrophobic interactions (representative) are shown as the red dashed line. Hydrophobic residues are shown as a red color.

*Interaction of **BrPQ5** with the beta5 subunit (chymotrypsin-like) of the human 20S proteasome*: **BrPQ5** appeared to occupy the S1 subpocket that accommodates small hydrophobic residues such as the Ala, Val, or Tyr of the substrate. **BrPQ5** showed three H-bonding interactions. The amino hydrogen of **BrPQ5** interacted with the side-chain hydroxy oxygen of Thr21. The quinone oxygen with a side-chain hydroxy hydrogen interacted with the amino hydrogen of Thr1. The quinone ring of **BrPQ5** interacted with Thr1, which is further stabilized by the π–π interaction of the quinone ring with Tyr169. The phenyl group of **BrPQ5** showed hydrophobic interactions with Gly47, Gly48, Ala49, and Ala50. Figure 5 shows a 2D interaction plot for the beta-5 subunit with **BrPQ5**.

**Figure 6:**
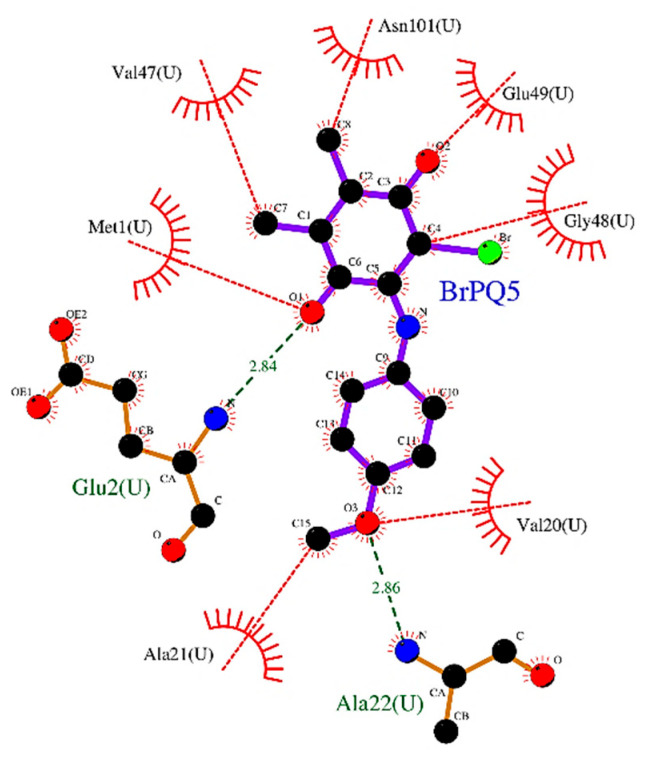
2D plot of **BrPQ5** in complex with beta2 subunit of human 20S proteasome. Atoms are colored by atom type. Bonds of ligand are shown in violet lines. Bonds of amino acids are shown as orange lines. H-bonds are shown as green dashed lines. Hydrophobic interactions (representative) are shown as a red dashed line. Hydrophobic residues are shown as a red color.

*Interaction of **BrPQ5** with the beta-2 subunit (trypsin-like) of the human 20S proteasome*: The acidic S1 subpocket, meant for accommodating basic P1 amino acids in the substrate, was found to interact well with **BrPQ5**. The quinone oxygen of **BrPQ5** showed an H-bonding interaction with the backbone NH of Glu2, while methoxy oxygen displayed an H-bonding interaction with the backbone NH of Ala22. A π–π interaction was observed. A hydrophobic interaction was observed with Met1, Val20, Ala21, Val47, Gly48, Glu49, and Asn101. A 2D interaction plot illustrating the complex of the beta-2 subunit with **BrPQ5** is shown in Figure 6.

**Figure 7:**
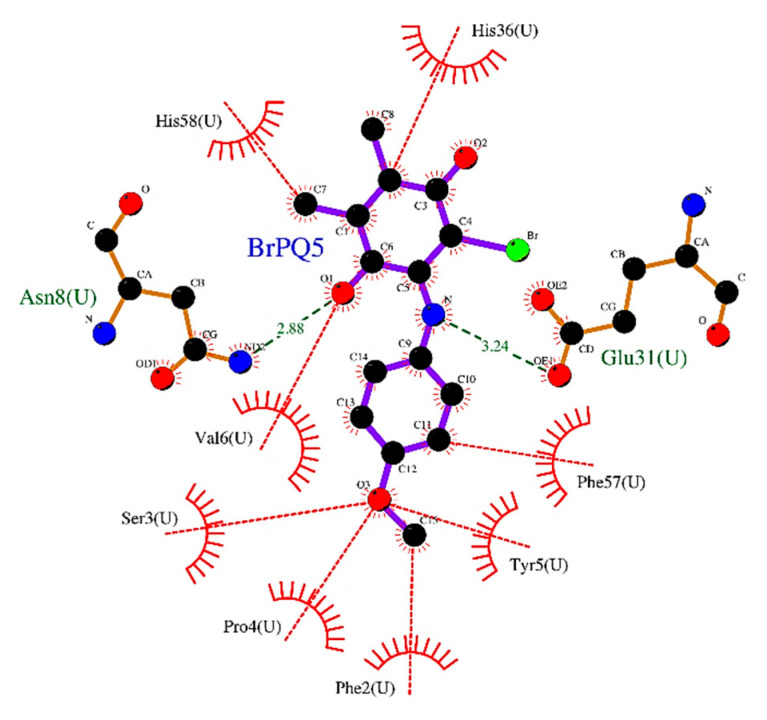
2D plot of **BrPQ5** in complex with the beta-1 subunit of the human 20S proteasome. Atoms are colored by atom type. Bonds of ligand are shown as violet lines. Bonds of amino acid are shown as orange lines. H-bonds are shown as green dashed lines. Hydrophobic interactions (representative) are shown as red dashed lines. Hydrophobic residues are shown as a red color.

*Interaction of **BrPQ5** with the beta-1 subunit (caspase-like) of the human 20S proteasome*: **BrPQ5** occupied the basic S1 subpocket meant for accommodating acidic amino acids. The quinone oxygen of **BrPQ5** formed an H-bonding interaction with the side-chain amide NH of Asn8, while amino NH formed an H-bonding interaction with the side-chain carbonyl oxygen of Glu31. Both the quinone ring and phenyl ring of **BrPQ5** exhibited a π–π interaction with Phe2 and His58, respectively. The interaction of the beta-1 subunit with **BrPQ5** is shown as a 2D interaction plot in Figure 7.

#### 2.2.5. In Vitro Anticancer Activity

**Figure 8:**
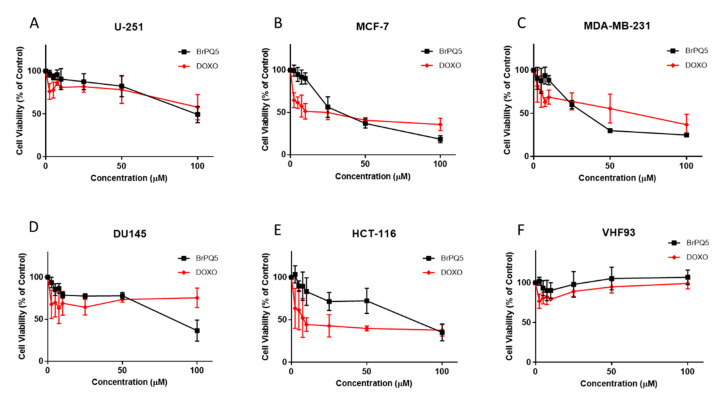
Cytotoxic evaluation of **BrPQ5** on U-251 glioma (**A**), MCF-7 (**B**), MDA-MB-231 breast cancers (**C**), DU145 prostate cancer (**D**), HCT-116 colon cancer (**E**), and VHF93 fibroblast (**F**) cell lines after 24 h treatment by MTT assay. The values are expressed as the mean ± SD.

To assess the anticancer activity of **BrPQ5**, U-251 human glioblastoma, MCF-7 and MDA-MB-231 human breast cancers, DU-145 human prostate cancer, and HCT-116 human colon cancer cell lines were used. These cell lines were chosen for in vitro studies according to NCI’s five dose–response analysis protocols. The VHF93 human fibroblast cell line was used as a noncancerous cell line to determine the specific anticancer activity of **BrPQ5**. As a positive control, doxorubicin HCl (DOXO) was used, containing the 1,4-quinone moiety. It is widely used to treat several cancer types [41]. According to the MTT cytotoxicity assay results, within the concentration range used in the study, **BrPQ5** showed cytotoxic activity in all cancer cell lines (Figure 8). The concentration–response curves and the 50% inhibitory concentration (IC_50_) values were generated using the inhibitor-normalized response variable slope function in GraphPad Prism 7 software. The lowest activity was against the U-251 human glioblastoma cell line, and the IC_50_ value could not be determined. DOXO had a similar cytotoxic profile with **BrPQ5** against the U-251 human glioblastoma cell line (Table 2). **BrPQ5** showed the highest cytotoxic activities against MCF-7 and MDA-MB-231 human breast cancer cell lines, and the IC_50_ values were 33.57 μM ± 1.7 and 33.65 μM ± 2.2, respectively (Figure 8B,C and Table 2). The IC_50_ values of DOXO were 17.52 μM ± 2.6 and 44.66 μM ± 9.8 against MCF-7 and MDA-MB-231 cells, respectively.

**BrPQ5** was less potent against DU-145 human prostate cancer and HCT-116 human colon cancer cells. The IC_50_ values for **BrPQ5** were 83.89 μM ± 12.8 and 74.33 μM ± 11, while IC_50_ values for DOXO were >100 μM and 12.84 μM ± 4.5, respectively. Promisingly, **BrPQ5** did not show cytotoxic activity against VHF93 human fibroblast cells, and the IC_50_ value could not be determined (Figure 8F). These results indicate high specificity of **BrPQ5** to cancer cells, similar to DOXO. According to the cytotoxicity assay results, MCF-7 cells were selected, and three different concentrations were chosen below the IC_50_ value (5, 10, 25 μM) for further studies.

**Figure 9:**
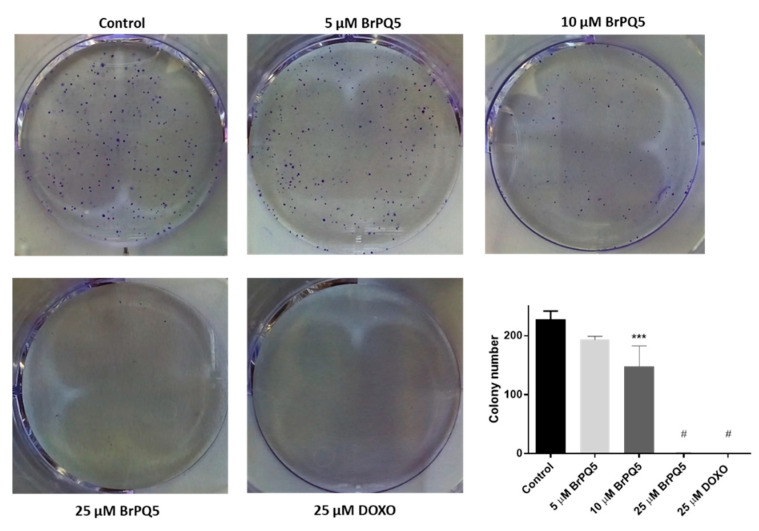
The effect of **BrPQ5** on MCF-7 cell proliferation by colony formation assay. (**A**). Representative images of colony formation assay. (**B**). Plotted mean values of the assay results. The values are expressed as the mean ± SD. (*** *p* < 0.001, ^#^ *p* < 0.0001 compared to control).

The clonogenic assay or colony formation assay is a widely used method to study the capacity of cancer cells growing from a single cell to a colony, and it provides data regarding the antiproliferative effect of test compounds [42]. The effect of **BrPQ5** on MCF-7 cell proliferation was assessed using a colony formation assay (Figure 9). **BrPQ5** significantly inhibited colony formation in a dose-dependent manner, and colony formation completely stopped at a 25 μM dose.

**Figure 10:**
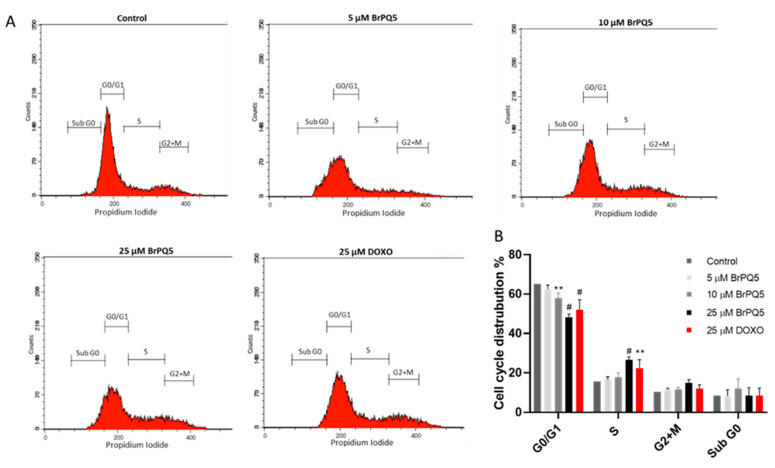
Cell cycle progression of MCF-7 cells treated with **BrPQ5** for 24 h and analyzed by flow cytometry. (**A**). Representative flow cytometry plots. (**B**). Plotted mean values of the assay results. The values are expressed as the mean ± SD. (** *p* < 0.01, ^#^ *p* < 0.0001 compared to control).

These results suggest strong antiproliferative activity of **BrPQ5** against MCF-7 breast cancer cells. Therefore, we further explored the cell proliferation changes by flow cytometric cell cycle analysis. The cell cycle analysis results suggested that **BrPQ5** treatment caused a concentration-dependent decrease in the G0/G1 phase, accompanied by concentration-dependent induction in the S phase (Figure 10). The S phase of the cell cycle represents the phase in which the cells replicate their DNA. In this case, the inhibition in the cell proliferation by **BrPQ5** in MCF-7 cells has been shown with MTT and colony formation assays. Thus, the increased cell population in the S phase seems unlikely to be related to increased DNA replication. Instead, it more likely suggests an accumulation in the S phase of the cell cycle with **BrPQ5** treatment. Additionally, a slight insignificant increase was observed in the G2/M. DOXO showed similar effects to **BrPQ5** on the cell cycle phases, and a reduction in the G0/G1 cell population with DOXO was suggested by other studies [43].

**Figure 11:**
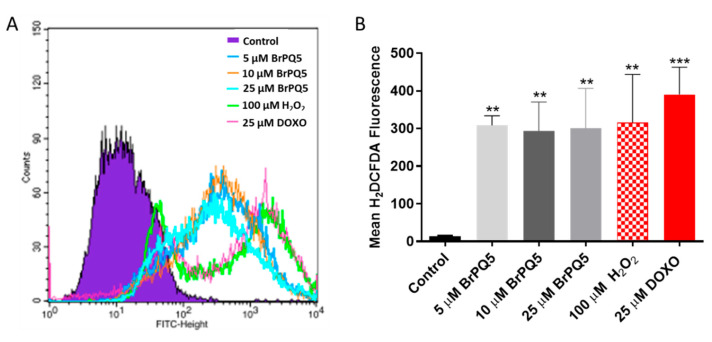
Oxidative stress changes of MCF-7 cells after **BrPQ5** treatment. The cells were analyzed by flow cytometry. (**A**). Representative flow cytometry plots (**B**). Plotted mean values of the assay results. The values are expressed as the mean ± SD. (** *p* < 0.01, *** *p* < 0.001 compared to control).

An antioxidant defense system within cells maintains a balance between reactive oxygen species (ROS) production and scavenging. Excessive ROS production triggers se- veral structural and molecular modifications and causes a cytotoxic effect [44]. Disruption in the redox homeostasis causes severe damage to cancer cells due to their high metabolic rate. Therefore, ROS-inducing therapeutic strategies for cancer treatment are gaining significant attention [45]. For this reason, the effect of **BrPQ5** on oxidative stress was investigated in this study. Intracellular ROS oxidizes H_2_DCFDA dye to generate a fluorescent product, and the intensity of the dye can then be detected by flow cytometry [46]. As shown in Figure 11, **BrPQ5** dramatically increased the oxidative stress level in all concentrations. As two known oxidative stress inducers, H_2_O_2_ and a prooxidant anticancer agent DOXO were used to compare the results. As indicated in Figure 11, the increment in the ROS production by **BrPQ5** was similar to H_2_O_2_ and DOXO. This suggests that the generation of oxidative stress can be an underlying mechanism responsible for the promising cytotoxic activity of **BrPQ5** against MCF-7 cells.

**Figure 12:**
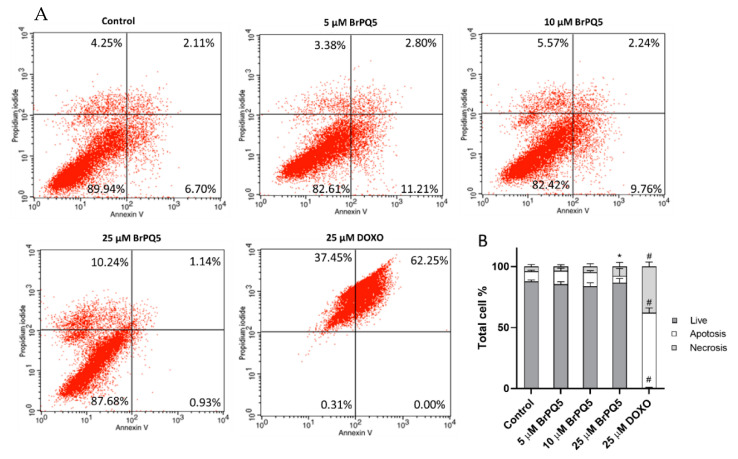
Apoptosis and necrosis evaluation of MCF-7 cells after **BrPQ5** treatment by flow cytometry. (**A**). Representative flow cytometry plots. (**B**). Plotted mean values of the assay results. The values are expressed as the mean ± SD. (* *p* < 0.05, ^#^ *p* < 0.0001 compared to control).

Furthermore, **BrPQ5** was investigated for its necrotic and apoptotic effects using flow cytometry. Unexpectedly, **BrPQ5** did not induce apoptosis, while a significant induction in the necrotic cell percentage was observed only with the highest concentration (25 μM) tested (Figure 12). In contrast, DOXO caused a significant increase in the apoptosis and necrosis rates. These results indicate that the apoptotic pathway was not involved in the cytotoxic activity of **BrPQ5**.

In summary, 25 µM **BrPQ5** was the highest tested concentration, which was lower than the IC_50_ value in MCF7 cells. Supportively, as can be seen in cytotoxicity data, 5 and 10 µM **BrPQ5** caused similar cytotoxic activity, while cytotoxicity increased with 25 µM **BrPQ5**. Also, these results were similar to the cell cycle and apoptosis/necrosis assay results. However, **BrPQ5** modulated ROS production in a dose-independent manner and caused significant ROS production at 5 µM, suggesting its ability to induce oxidative stress in low concentrations. It is known that cancer cells can adapt themselves to low levels of ROS to continue tumor development, and only a powerful ROS induction can cause cell effective cell damage [47]. Thus, it is thought that a powerful ROS burst with 5 µM **BrPQ5** treatment may be responsible for the antiproliferative effect in MCF7 cells. The changes in the cell cycle and cell necrosis with increased concentrations of **BrPQ5** contri-buted to the dose-dependent antiproliferative effects in MCF7 cells.

**Figure 13:**
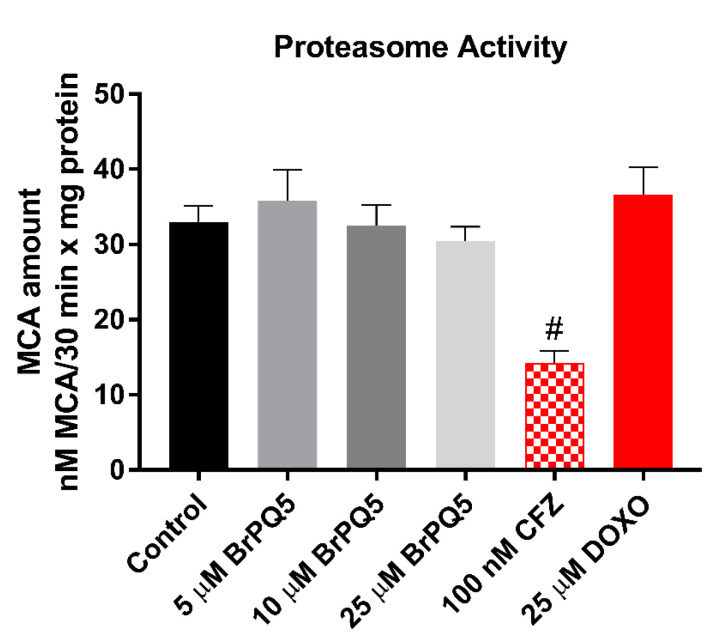
Proteasome activity evaluation of MCF-7 cells after **BrPQ5** treatment. The values are expressed as the mean ± SD. CFZ: Carfilzomib, (^#^ *p* < 0.0001 compared to control).

In the anticancer research associated with quinone chemistry, several 1,4-quinone molecules were developed and have demonstrated proteasome inhibition [48,49,50]. In light of these papers and our in silico study results that indicated **BrPQ5**’s interaction with proteasome catalytic subunits, we focused on further investigating the **BrPQ5** effects on the catalytic activity of the proteasome. Especially, the β5 catalytic subunit of the proteasome is the main target for cancer treatment, and there are several clinically used proteasome inhibitors on the market, such as bortezomib, carfilzomib, and ixazomib [51]. Our results showed that **BrPQ5** and DOXO did not inhibit the proteasome activity, while 100 nM carfilzomib treatment caused significant proteasome inhibition (Figure 13). Based on these insights, it can be interpreted that **BrPQ5** interaction with proteasome β5 catalytic subunit, as determined using in silico experiments, did not inhibit the β5 subunit catalytic activity.

## 3. Conclusions

Herein, the brominated PQ analogs (**BrPQ1–10**) functionalized in amino phenyl moiety were resynthesized and evaluated for their antiproliferative effects in terms of their inhibition efficiency. The preliminary in vitro antiproliferative activities of all BrPQ analogs against the full NCI 60 cell line panel was determined by NCI at a 10 μM concentration. The in vitro preliminary antiproliferative evaluation revealed that the BrPQ analogs with alkoxy substituent(s) in the amino phenyl moiety have maximum sensitivity toward many cancer cell lines, particularly CCRF-CEM, HL-60(TB), K-562, MOLT-4, and SR leukemia cell lines, as well as breast cancer cell lines. **BrPQ5** was the most potent against leukemia and breast cancer cell lines and was selected for five-dose NCI screening. Based on this preliminary one-dose and five-dose antiproliferative screening, further studies are required to provide more insights into the biological mechanism of action. Thus, in vitro studies were performed with different cancer cell models. The findings from these analyses hinted at the promising anticancer activity of **BrPQ5** against the MCF-7 breast cancer cell line with the absence of any significant cytotoxicity against noncancerous VHF93 human fibroblast cells. Further studies were performed with **BrPQ5** on MCF-7 cells to compare it with DOXO, a well-known chemotherapeutic drug. **BrPQ5** treatment caused a strong oxidative stress induction with all tested concentrations and caused dose-dependent cytotoxic activity and the inhibition of cell proliferation in MCF7 cells. Oxidative stress induction is accompanied by cell cycle arrest and cellular necrosis in the highest concentration. Accordingly, these were the key pathways eliciting the anticancer activity of **BrPQ5** in the MCF-7 cells. Assertively, these effects were similar to DOXO, which highlights the therapeutic potential of **BrPQ5** in the treatment of breast cancer. **BrPQ5** did not significantly inhibit the catalytic activity of the proteasome, and the proteasomal pathway did not play a role in the anticancer activity of **BrPQ5** in the MCF-7 cells as suggested by in silico studies. The peptidomimetic inhibitors such as carfilzomib and ONX-0914 tend to occupy all four subpockets (S1–S4) meant for accommodating P1–P4 residues. In contrast, bortezomib and IPSI-001 occupied three (S1–S3) and two (S1 and S2) subpockets, respectively [52]. Our compound **BrPQ5**, when compared to reported inhibitors, is quite small and could interact only with residues lining the S1 subpocket in all three cases (beta-5: S1 hydrophobic, beta-2: S1: acidic, and beta-1: S1 basic). Irrespective of the nature of the pocket, **BrPQ5** has shown favorable interactions in all three subunits. However, it appears that these interactions are insufficient to retain this relatively small molecule in the active site in the absence of no interacting pharmacophoric features with other subpockets. This may be why these compounds fail to show any significant inhibition in the proteasome inhibition assay. Extending the molecular architecture with minimal pharmacophoric features to interact with the S2 subpocket may provide us with a suitable candidate. However, favorable in silico ADME predictions in terms of drug-likeness, oral bioavailability, and pharmacokinetic parameters suggested that the brominated BrPQ analogs could serve as promising hit and/or lead analogs for the discovery of new compounds with more effective proteasome inhibiting activities for future studies. In our future studies, we plan to further design and synthesize similar structures. More efforts are currently underway in our laboratory to generate new targeted libraries.

## 4. Materials and Methods

The molecules investigated within this research have been synthesized by our group [25,28]. Molecular docking simulation was carried out on an HP desktop with an Intel^®^ Core™ i7-6700 CPU @ 3.40GHz × 8 processor, Intel^®^ HD Graphics 530 (SKL GT2) Graphics, 7.7 GB Memory, and 1 TB disk capacity. Programs and software were installed on Ubuntu 20.04.3 LTS, 64-bit, 3.36.8 version, and X11 windowing system. The software and webserver used for the simulation and analysis included ChemDraw 19.1, UCSF Chimera, MGLTools-1.5.7, Autodock-4.2, LigPlot+, and pdb2pqr server.

### 4.1. Biological Evaluation

#### 4.1.1. In Vitro Antiproliferative Activity at a One-Dose Concentration by NCI

Three different series of the brominated PQ analogs were submitted to the National Cancer Institute (NCI), Bethesda, USA. As per the standard protocol of NCI, all compounds were evaluated for their antiproliferative activity at a single-dose assay (10 µM concentration in DMSO) on a panel of 60 cancer cell lines derived from leukemia, non-small cell lung, colon, CNS, melanoma, ovarian, renal, prostate, and breast cancer as per protocol. Tested compounds were added to the microtiter culture plates, followed by incubation for 48 h at 37 °C. Sulforhodamine B (SRB), a protein-binding dye, was used for endpoint determination. The percentage growth of the treated cells was determined and compared to the untreated control cells, and the results of each tested compound were reported. Data from the one-dose experiments pertain to the percentage growth at 10 μM [34,35,53].

#### 4.1.2. In Vitro Antiproliferative Activity at a Five-Dose Concentration by NCI

Serial 5 × 10-fold dilutions from an initial DMSO stock solution were performed before incubation at each concentration. The most promising BrPQ analog (**BrPQ5**) was then elevated by DTP-NCI for a higher testing level to determine three dose–response parameters (GI50, TGI, and LC50) for each cell line after establishing a dose–response curve from five different concentrations of 0.01, 0.1, 1, 10, and 100 µM for BrPQ5. The detailed procedure for the latter assay had been elaborated earlier [35,53,54].

#### 4.1.3. Cell Culture and Cytotoxicity Assay

U-251 human glioblastoma, MCF-7, MDA-MB-231 human breast cancers, DU-145 human prostate cancer, HCT-116 human colon cancer, and VHF93 human fibroblast cell lines were obtained from the American Type Culture Collection. U-251, MDA-MB-231, DU-145, HCT-116, and VHF93 cells were grown in DMEM (Gibco), and MCF-7 cells were cultured in DMEM:F12 (Gibco). The culture medium was supplemented with 10% heat-inactivated fetal bovine serum (Gibco), 10 U/mL penicillin, and 100 μg/mL streptomycin (Gibco) at 37 °C in a 5% CO_2_ humidified atmosphere. BrPQ5 and doxorubicin HCl (DOXO) were dissolved in DMSO. DOXO was used as a reference compound to compare the effects of BrPQ5. For all assays, 0.5% DMSO concentration was not exceeded for cell treatments, and the control group was treated with 0.5% DMSO.

Cell viability was assessed using the MTT assay (BioMatik). For this purpose, 1 × 10^4^ cells per well were seeded in 96 well plates and incubated overnight. The cells were then treated with increasing concentrations of compounds (2.5, 5, 7.5, 10, 25, 50, 100 μM) for 24 h. After the treatment, the medium was replaced with fresh media containing 1 mg/mL MTT dye. The cells were incubated for an additional 3 h for the formation of formazan by mitochondrial succinic dehydrogenase. Subsequently, the medium was removed, and the formazan crystals were dissolved in DMSO. The absorbance value of the dye was measured at 570 nm with a microplate reader (Enspire, PerkinElmer, Waltham, MA, USA). The IC_50_ values (compound concentrations that cause the 50% reduction in the cell viability) were calculated using GraphPad Prism 7 Software.

#### 4.1.4. Colony Formation Assay

The effect of **BrPQ5** on cell proliferation was assessed using a colony formation assay. Briefly, 1500 MCF-7 cells per well were seeded in 6-well plates and incubated overnight. The MCF-7 cells were treated with 5, 10, 25 μM **BrPQ5**, and 25 μM DOXO along with the control for 24 h. Then, the wells were washed with PBS, and the cells were grown in the fresh medium for 10 days. After the incubation period, the medium was removed, and the cells were fixed with cold methanol for 5 min. The cells were dyed with 0.5% crystal violet (10% methanol) for 20 min. Then, the dye was discarded, and the wells were washed with distilled water until the water became colorless. The plates were air-dried, and the wells were photographed under natural light. The colonies were manually counted from the photographs.

#### 4.1.5. Oxidative Stress Evaluation

The effect of **BrPQ5** on oxidative stress was analyzed with (5-(and-6)-chloromethyl-2′,7′-dichlorodihydrofluorescein diacetate (H_2_DCFDA) (Sigma, St. Louis, MO, USA) staining. Briefly, 3 × 10^5^ MCF-7 cells per well were seeded in 6-well plates and incubated overnight. The MCF-7 cells were treated with 5, 10, and 25 μM **BrPQ5** along with the control for 24 h. 25 μM DOXO and 100 μM H_2_O_2_ were used as positive controls. Subsequently, the cells were collected by trypsinization, and 20 μM of H_2_DCFDA dye was added to the cell suspension. The cells were incubated for 30 min and centrifuged at 500× *g* for 5 min. The supernatant was discarded, and the cells were suspended in PBS. The changes in oxidative stress with the treatments were analyzed by flow cytometry (BD Biosciences, San Jose, CA, USA).

#### 4.1.6. Cell Cycle Analysis

The effect of **BrPQ5** on cell cycle arrest was evaluated by the Muse Cell Cycle Kit (Millipore) according to the manufacturer’s protocol. The MCF-7 cells were treated with 5, 10, and 25 μM **BrPQ5** and 25 μM DOXO along with the control for 24 h. The cells were collected by trypsinization and fixed in ice-cold 70% ethanol for 3 h. Subsequently, the cells were collected by 300× *g* centrifugation for 5 min. The cell pellet was suspended in 200 μL assay buffer and incubated for 30 min in the dark. The differences in the cell cycle stages (G0/G1, S, G2+M, sub G0) were analyzed by flow cytometry (BD Biosciences).

#### 4.1.7. Apoptosis and Necrosis Analysis

The apoptotic and necrotic cell rate was analyzed with the Annexin V-FITC Apoptosis Detection Kit (Millipore) according to the manufacturer’s protocol. For the assay, the MCF-7 cells were treated with 5, 10, and 25 μM **BrPQ5** and 25 μM DOXO along with the control for 24 h. The cells were collected by 300× *g* centrifugation after trypsinization. The cells were resuspended in binding buffer and incubated with Annexin V-FITC and propidium iodide (PI) for 15 min at room temperature in the dark. The cells were centrifuged at 300× *g* for 5 min and resuspended in the binding buffer. The flow cytometric analysis was carried out immediately by BD Bioscience flow cytometry, and the percentages of apoptotic and necrotic cells were calculated using BD Bioscience software.

#### 4.1.8. Proteasome Activity

Proteasome β5 subunit activity was directly measured with the hydrolysis of the fluorogenic substrate Suc-Leu-Leu-Val-Tyr-AMC (Sigma-Aldrich). The MCF-7 cells were treated with 5, 10, and 25 μM **BrPQ5** and 25 μM DOXO along with control for 24 h. Then, the cells were scraped on ice, and cell lysates were prepared in lysis buffer (0.25 M saccharose, 25 mM HEPES, 10 mM MgCl_2_, 1 mM EDTA, 1 mM DTT) with three freeze–thaw cycles. The lysates were centrifuged (13,500 rpm, 30 min, 4 °C). The total protein concentration was determined using the Protein Assay Kit (Bio-Rad, Hercules, CA, USA). Free AMC concentration was used to create a standard curve for quantitative analysis. The proteasome activity was measured from the supernatants after 30 min incubation with the fluorogenic substrate in buffer containing 225 mM Tris, 45 mM potassium chloride, 7.5 mM magnesium acetate, 7.5 mM magnesium chloride, and 1 mM DTT, pH 7.8 using EnSpire multimode reader (PerkinElmer) at 360 nm excitation/460 nm emission.

#### 4.1.9. Statistics

Statistical analysis was applied using GraphPad Prism 7 software (GraphPad Software, La Jolla, California, USA). The data are expressed as the mean ± standard deviation (SD) of at least three independent experiments. Analysis of ANOVA variance with the Tukey’s *post hoc* test was used for multiple comparisons. The level of significance was set at *p* < 0.05.

### 4.2. In Silico Study

In this study, the free online server SwissADME (http://swissadme.ch/index, accessed on 15 May 2022) was used to determine the ADME and pharmacokinetic properties of the brominated BrPQ analog (**BrPQ5**). The chemical structure was drawn using MarvinSketch to generate SMILE and inserted directly on the webpage to initiate the prediction process. The different physical properties, pharmacokinetic parameters, and ADME parameters, along with the BOILED-Egg chart, were downloaded from the server and analyzed.

A molecular docking simulation was carried out using AutoDock-4.2 and MGLTools-1.5.7 [55]. The step-by-step procedure is provided below.

(i) *Protein preparation*: Coordinates of human 20S proteasome subunits beta-1 (chain M), beta-2 (chain K), and beta-5 (chain L) were manually extracted from the X-ray crystal structure of the human 20S proteasome in a complex with carfilzomib (PDB:4R67) [56]. The coordinates of subunits beta-1 and beta-2 were then superimposed on beta-5 and were rewritten using UCSF chimera [57]. This allowed us to use the coordinates of the co-crystallized ligand (3BV) of subunit beta-5 to be used for all three to specify the grid box. Then, all the three extracted chains were prepared for docking through the pdb2pqr server. Further, the non-polar hydrogens were merged, the AD4 atom type was assigned, and gasteiger charges were added using MGLTools-1.5.7. It was then saved as respective_protein.pdbqt.
(ii) *Ligand preparation*: The structure of **BrPQ5** was sketched in Chemdraw-19, 3D geometry optimized, energy minimized, and saved as ligand.pdb. Torsion and charge were assigned to the ligand and then saved as ligand.pdbqt using MGLTools-1.5.7.
(iii) *Docking preparation, run, analysis*: (a) Generation of grid map files: the grid over the binding site on respective_protein (*.pdbqt) was generated using the coordinates of carfilzomib (3BV) bound to subunit beta-5 (chain L). The center of the grid was specified as the center of the protein-bound ligand with a box dimension of 40:42:40 and grid spacing of 0.375 Å. Map types were set considering the atom types in the **BrPQ5**. The grid parameter file was then saved as respective_protein.gpf and used to generate map files using the autogrid4 execution file; (b) Docking: the docking parameter file for each subunit was then written for **BrPQ5** (respective_protein_BrPQ5.dpf) with 100 hybrid GA-LS runs, 150 population size, 2,500,000 energy evaluations, and 27,000 generations. The generated map files and the *.dpf files docking simulation were performed using the autodock4 execution file. (c) Analysis: the top scoring conformer in the largest cluster of the respective docking log file (*.dlg) was then picked for interaction analysis, and the 3D interaction plot was then saved as *.png (in Supplementary File). All these steps were carried out using MGLTools-1.5.7. Further 2D plots were also generated for each complex to better understand protein–ligand interactions using LigPlot+ [58]. The coordinates of respective_proteins, co-crystallized ligand and **BrPQ5**, respective_protein.gpf, and respective_protein_BrPQ5.dpf files used in the study are provided in the Supplementary File.

## Figures and Tables

**Table 1:** GI_50_, TGI, and LC_50_ values (in µM) of antiproliferative activity data as per a five-dose assay of the BrPQ analog (**BrPQ5**).

Molecule	**BrPQ5 (NCI: D-825197/1)**		
Panel/Cell Line	GI_50_	TGI	LC_50_
**Leukemia**			
CCRF-CEM	2.33	>100	>100
HL-60 (TB)	2.34	6.82	>100
K-562	2.25	>100	>100
MOLT-4	2.67	>100	>100
RPMI-8226	2.21	5.01	>100
SR	3.21	>100	>100
**Non-Small Cell Lung Cancer**			
A549/ATCC	18.70	45.70	>100
EKVX	1.69	3.12	5.77
HOP-62	15.00	38.60	99.70
HOP-92	1.55	3.08	6.10
NCI-H226	13.00	27.60	58.50
NCI-H23	4.41	23.70	>100
NCI-H322M	14.60	27.80	53.10
NCI-H460	18.80	42.60	96.10
NCI-H522	12.70	31.80	79.50
**Colon Cancer**			
COLO 205	21.30	51.10	>100
HCC-2998	12.40	26.50	56.50
HCT-116	1.76	3.37	6.46
HCT-15	11.40	27.70	67.40
HT29	27.40	79.60	>100
KM12	5.02	19.00	58.00
SW-620	2.07	4.54	9.97
**CNS Cancer**			
SF-268	11.90	28.20	67.00
SF-295	13.80	27.00	52.80
SF-539	13.50	26.70	52.90
SNB-19	10.50	22.80	49.10
SNB-75	11.00	23.50	50.20
U251	19.30	3.98	8.24
**Melanoma**			
LOX IMVI	1.67	3.17	5.99
MALME-3M	14.50	28.30	55.20
M14	11.70	24.80	52.60
MDA-MB-435	1.91	3.49	6.36
SK-MEL-2	11.40	28.30	70.60
SK-MEL-28	2.30	4.76	9.88
SK-MEL-5	13.10	26.00	51.50
UACC-257	1.75	3.37	6.51
UACC-62	7.74	20.70	46.80
**Ovarian Cancer**			
IGROV1	1.80	3.39	6.38
OVCAR-3	1.72	3.13	5.72
OVCAR-4	1.60	2.97	5.49
OVCAR-5	5.07	21.80	75.40
OVCAR-8	2.22	5.31	>100
NCI/ADR-RES	2.04	4.65	>100
SK-OV-3	12.30	26.10	55.00
**Renal Cancer**			
786-0	15.70	29.90	56.90
A498	1.62	33.60	>100
ACHN	2.87	8.76	29.40
CAKI-1	1.72	3.11	5.63
RXF 393	2.71	8.13	32.30
SN12C	11.00	23.20	49.10
TK-10	25.20	40.90	66.30
UO-31	1.73	3.16	5.77
**Prostate Cancer**			
PC-3	2.78	9.05	33.50
DU-145	15.00	29.10	56.50
**Breast Cancer**			
MCF7	1.78	3.77	7.98
MDA-MB-231/ATCC	1.88	3.96	8.34
HS 578T	11.80	57.50	>100
BT-549	11.80	24.40	50.40
T-47D	1.93	4.81	>100
MDA-MB-468	1.56	3.16	6.40

**Table 2:** IC_50_ values of **BrPQ5**, on U-251 glioma, MCF-7 and MDA-MB-231 breast cancers, DU145.

Cell Lines	IC_50_± (µM)	
	BrPQ5	DOXO
**U-251**	>100	>100
**MCF-7**	33.57 ± 1.7	17.52 ± 2.6
**MDA-MB-231**	33.65 ± 2.2	44.66 ± 9.8
**DU-145**	83.89 ± 12.8	>100
**HCT-116**	74.33 ± 11	12.84 ± 4.5
**VHF93**	>100	>100

IC_50_ values were calculated with MTT assay after 24 h **BrPQ5** treatment. IC_50_±: The compound concentration required to inhibit cell viability by 50%. The values are expressed as the mean ± SD. Prostate cancer, HCT-116 colon cancer, and VHF93 fibroblast cell lines.

## Data Availability

Data is contained within the article and Supplementary Material.

## Supplementary Materials

The following supporting information can be downloaded at: https://www.mdpi.com/article/10.3390/ph15070777/s1, Figure S1: Purity chromatogram of the **BrPQ1**; Figure S2: Purity chromatogram of the **BrPQ2**; Figure S3: Purity chromatogram of the **BrPQ3**; Figure S4: Purity chromatogram of the **BrPQ4**; Figure S5: Purity chromatogram of the **BrPQ5**; Figure S6: Purity chromatogram of the **BrPQ6**; Figure S7: Purity chromatogram of the **BrPQ7**; Figure S8: Purity chromatogram of the **BrPQ8**; Figure S9: Purity chromatogram of the **BrPQ9**; Figure S10: Purity chromatogram of the **BrPQ10**; Figure S11: Single-dose in vitro antiproliferative activity of **BrPQ1**; Figure S12: Single-dose in vitro antiproliferative activity of **BrPQ2**; Figure S13: Single-dose in vitro antiproliferative activity of **BrPQ3**; Figure S14: Single-dose in vitro antiproliferative activity of **BrPQ4**; Figure S15: Single-dose in vitro antiproliferative activity of **BrPQ5**; Figure S16: Single-dose in vitro antiproliferative activity of **BrPQ6**; Figure S17: Single-dose in vitro antiproliferative activity of **BrPQ7**; Figure S18: Single-dose in vitro antiproliferative activity of **BrPQ8**; Figure S19: Single-dose in vitro antiproliferative activity of **BrPQ9**; Figure S20: Single-dose in vitro antiproliferative activity of **BrPQ10**; Table S1. Antiproliferative activity data as per single dose assay at 10 µM concentration as percent cell growth of the selected hybrid molecules; Figure S21: Dose response curves of five-dose in vitro antiproliferative activity of **BrPQ5**; Figure S22: Five-dose in vitro antiproliferative activity of **BrPQ5**; Figure S23: Data of five-dose in vitro antiproliferative activity of **BrPQ5**; Figure S24. The physicochemical properties and pharmacokinetic profile of the BrPQ analog (**BrPQ5**) evaluated using SwissADME; Figure S25. The generated BOILED-Egg graph of the BrPQ analog (**BrPQ5**) using SwissADME; Figure S26. The heatmap illustrating the five-dose in vitro antiproliferative activity of **BrPQ5**.
