# Supplementary material for: Exploring the Anticancer Effects of Brominated Plastoquinone Analogs with Promising Cytotoxic Activity in MCF-7 Breast Cancer Cells via Cell Cycle Arrest and Oxidative Stress Induction
Source: Pharmaceuticals (Basel). 2022 Jun 22;15(7):777. doi: 10.3390/ph15070777 (PMC9318129; doi:10.3390/ph15070777)
Supplement: Supplementary file 1 [file pharmaceuticals-15-00777-s001.zip › pharmaceuticals-1734064-supplementary.pdf]

Supplemental Material

Exploring the Anticancer Effects of Brominated Plastoquinone Analogs with Promising Cytotoxic Activity in MCF-7 Breast Cancer Cells *via* Cell Cycle Arrest and Oxidative Stress Induction

Ayse Tarbin Jannuzzi [1,†], Ayse Mine Yilmaz Goler [2,3,†], Nilüfer Bayrak [4], Mahmut Yıldız [5], Hatice Yıldırım [4], Betul Karademir Yilmaz [2,3], Deepak Shilkar [6], Raghusrinivasan Jayaprakash Venkatesan [7], Venkatesan Jayaprakash [6] and Amaç Fatih TuYuN [8,*]

[1] Department of Pharmaceutical Toxicology, Faculty of Pharmacy, Istanbul University, Beyazit, Istanbul 34116, Turkey; tarbin.cevik@istanbul.edu.tr
[2] Department of Biochemistry, School of Medicine, Marmara University, Istanbul 34854, Turkey; ayse.mine@marmara.edu.tr (A.M.Y.G.); betulkarademir@marmara.edu.tr (B.K.Y.)
[3] Genetic and Metabolic Diseases Research and Investigation Center, Marmara University, Istanbul 34854, Turkey
[4] Department of Chemistry, Faculty of Engineering, Istanbul University-Cerrahpasa, Avcılar, Istanbul 34320, Turkey; nbayrak@istanbul.edu.tr (N.B.); hyildirim@iuc.edu.tr (H.Y.)
[5] Chemistry Department, Gebze Technical University, Gebze, Kocaeli 41400, Turkey; yildizm@gtu.edu.tr
[6] Department of Pharmaceutical Sciences & Technology, Birla Institute of Technology, Mesra, Ranchi 835215, India; deepakshilkar@live.com (D.S.); venkatesanj@bitmesra.ac.in (V.J.)
[7] Department of Industrial and Systems Engg, Indian Institute of Technology, Kharagpur 721302, West Bengal, India; rover.rags@gmail.com
[8] Department of Chemistry, Faculty of Science, Istanbul University, Fatih, Istanbul 34126, Turkey
* Correspondence: aftuyun@gmail.com or aftuyun@istanbul.edu.tr; Tel.: +90-212-440-0000
† These authors contributed equally to this work.

Contents

Purity Chromatograms of the Brominated PQ Analogs (**BrPQ1-10**) S3-7

Single-Dose *in vitro* Antiproliferative Activity Data (**BrPQ1-10**) S8-17

Antiproliferative Activity Data as per Single Dose Assay (Table) S18-20

Five-Dose *in vitro* Antiproliferative Activity Data (**BrPQ5**) S21-23

The Physicochemical Properties and Pharmacokinetic Profile of **BrPQ5** S24

The Generated BOILED-Egg Graph of **BrPQ5** S25

The heatmap illustrating the five-dose *in vitro* antiproliferative activity of **BrPQ5** S26

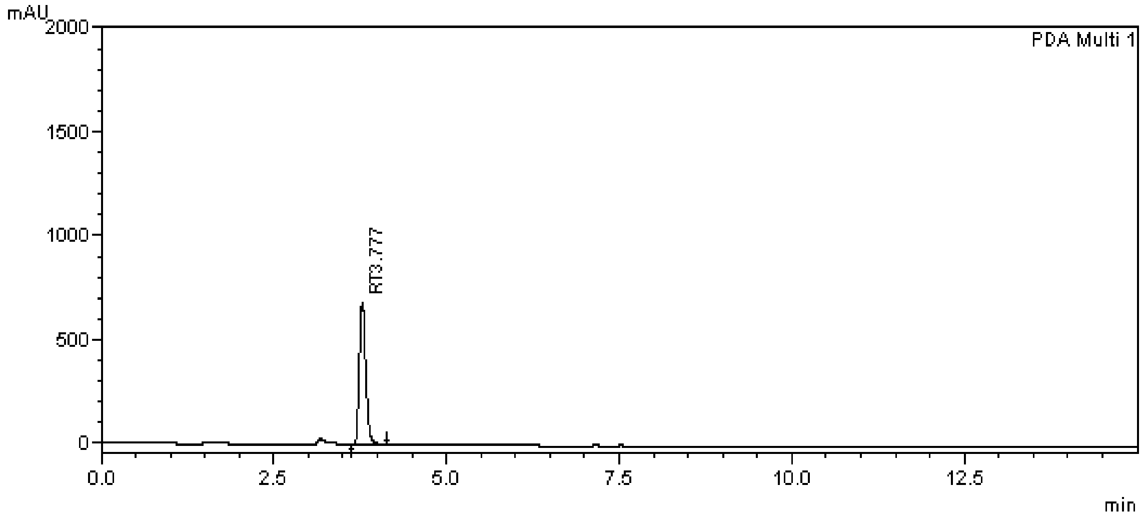

1 PDA Multi 1

<Results>

PDA Ch1 215nm 4nm

Peak#	Ret. Time	Area	Height	Area %	Height %	Resolution
1	3.777	4457266	682189	100.000	100.000	0.000
Total		4457266	682189	100.000	100.000	

Figure S1: Purity chromatogram of the **BrPQ1**

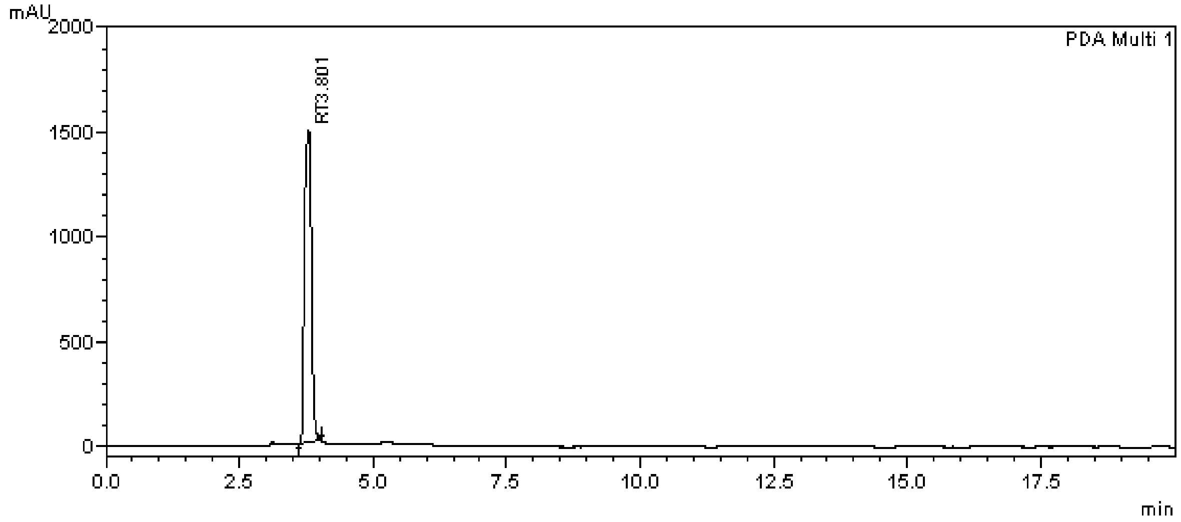

1 PDA Multi 1

<Results>

PDA Ch1 215nm 4nm

Peak#	Ret. Time	Area	Height	Area %	Height %	Resolution
1	3.801	13398587	1482117	100.000	100.000	0.000
Total		13398587	1482117	100.000	100.000	

Figure S2: Purity chromatogram of the **BrPQ2**

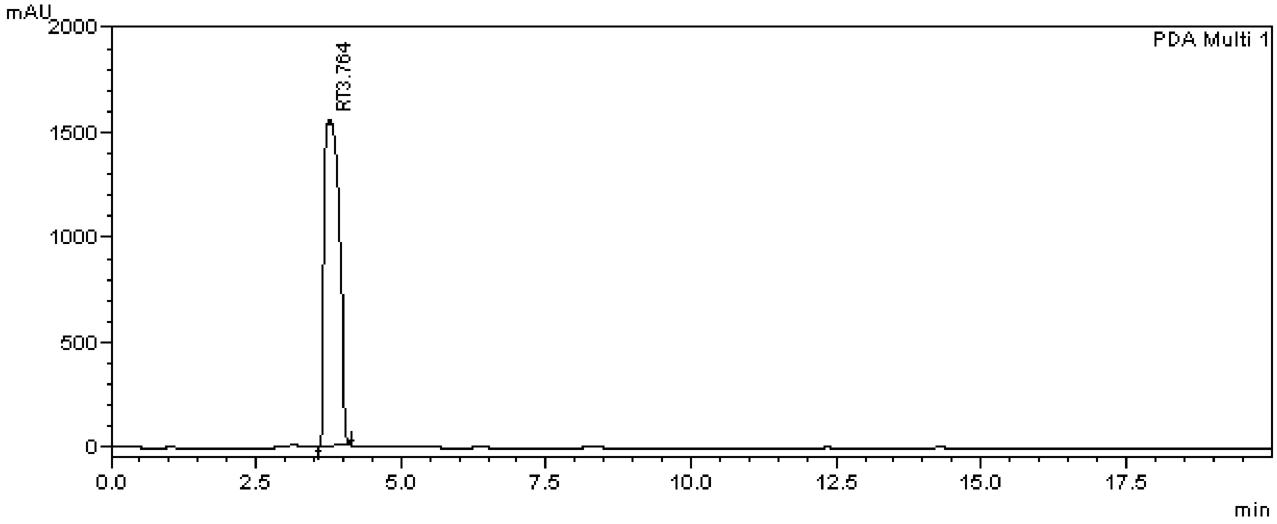

1 PDA Multi 1

<Results>

PDA Ch1 215nm 4nm

Peak#	Ret. Time	Area	Height	Area %	Height %	Resolution
1	3.764	27973200	1553336	100.000	100.000	0.000
Total		27973200	1553336	100.000	100.000	

Figure S3: Purity chromatogram of the **BrPQ3**

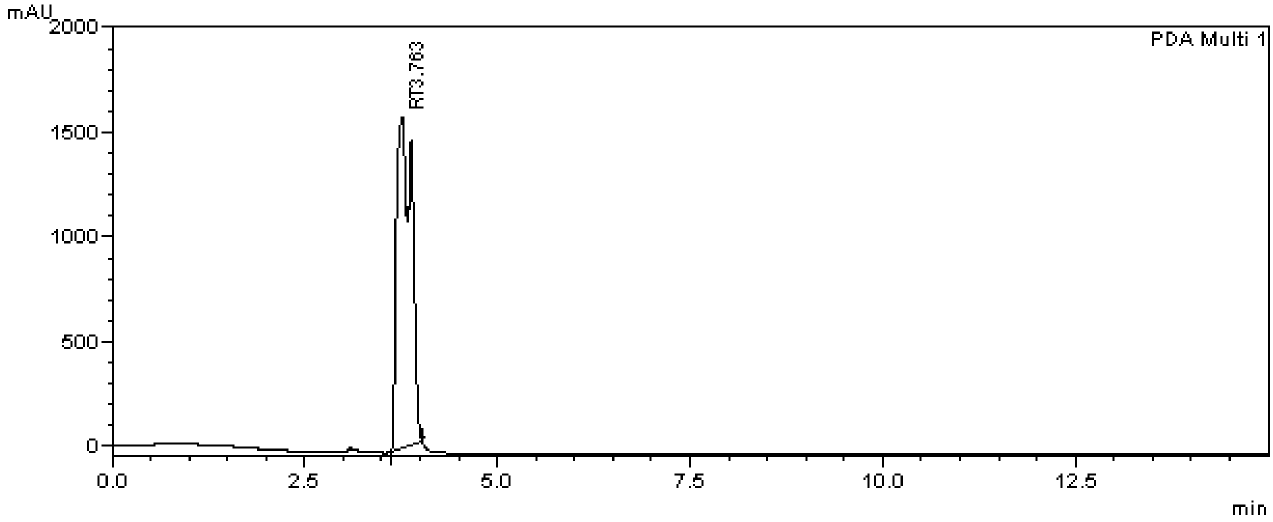

1 PDA Multi 1

<Results>

PDA Ch1 215nm 4nm

Peak#	Ret. Time	Area	Height	Area %	Height %	Resolution
1	3.763	21541079	1578997	100.000	100.000	0.000
Total		21541079	1578997	100.000	100.000	

Figure S4: Purity chromatogram of the **BrPQ4**

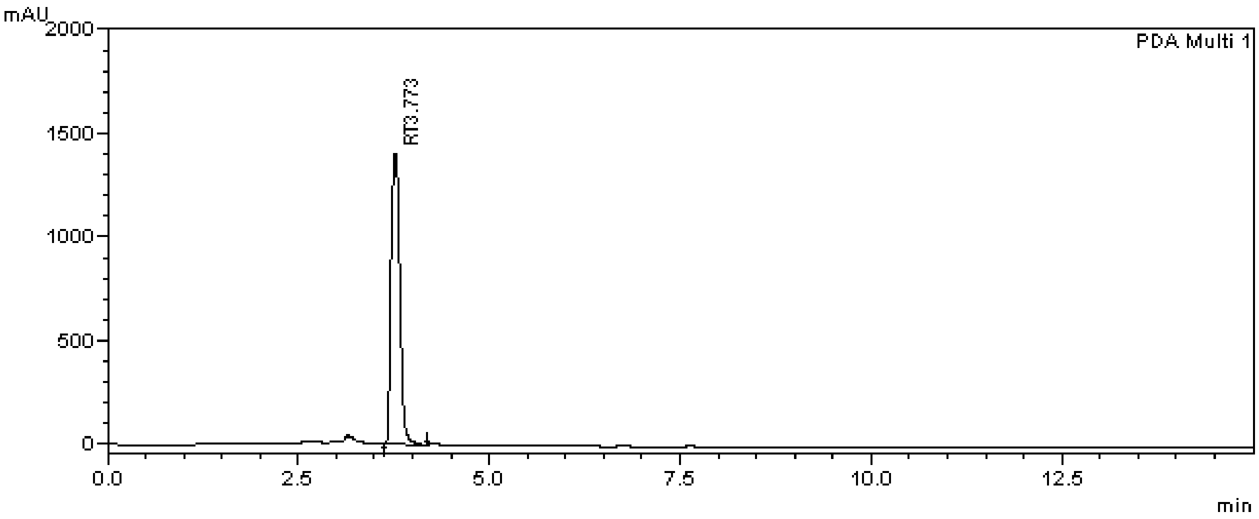

1 PDA Multi 1

<Results>

PDA Ch1 215nm 4nm

Peak#	Ret. Time	Area	Height	Area %	Height %	Resolution
1	3.773	11024909	1403620	100.000	100.000	0.000
Total		11024909	1403620	100.000	100.000	

Figure S5: Purity chromatogram of the **BrPQ5**

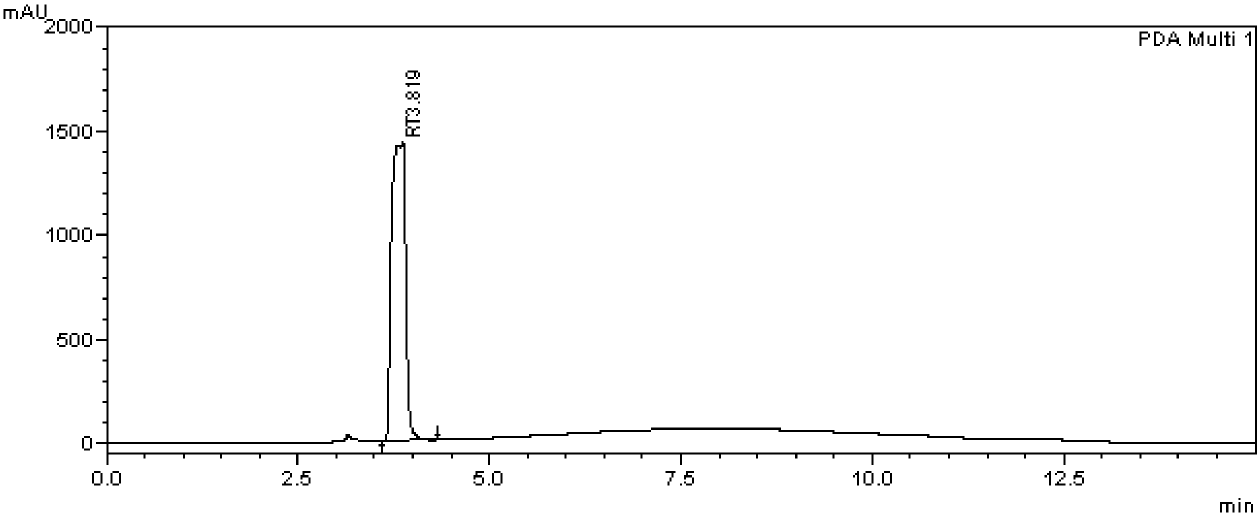

1 PDA Multi 1

<Results>

PDA Ch1 215nm 4nm

Peak#	Ret. Time	Area	Height	Area %	Height %	Resolution
1	3.819	18175414	1403416	100.000	100.000	0.000
Total		18175414	1403416	100.000	100.000	

Figure S6: Purity chromatogram of the **BrPQ6**

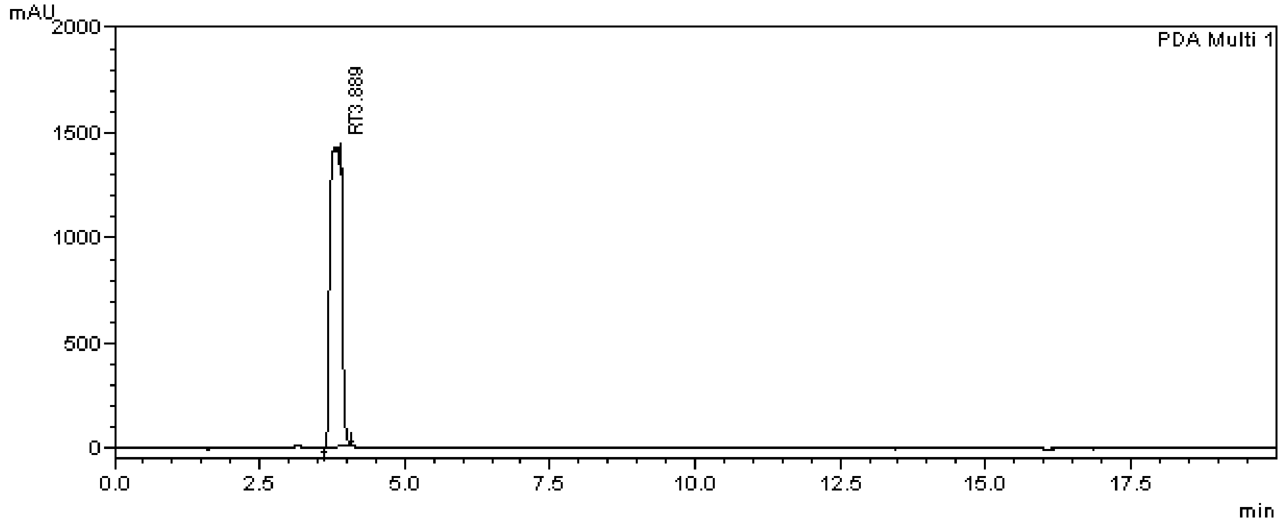

1 PDA Multi 1

<Results>

PDA Ch1 215nm 4nm

Peak#	Ret. Time	Area	Height	Area %	Height %	Resolution
1	3.889	20454716	1437504	100.000	100.000	0.000
Total		20454716	1437504	100.000	100.000	

Figure S7: Purity chromatogram of the **BrPQ7**

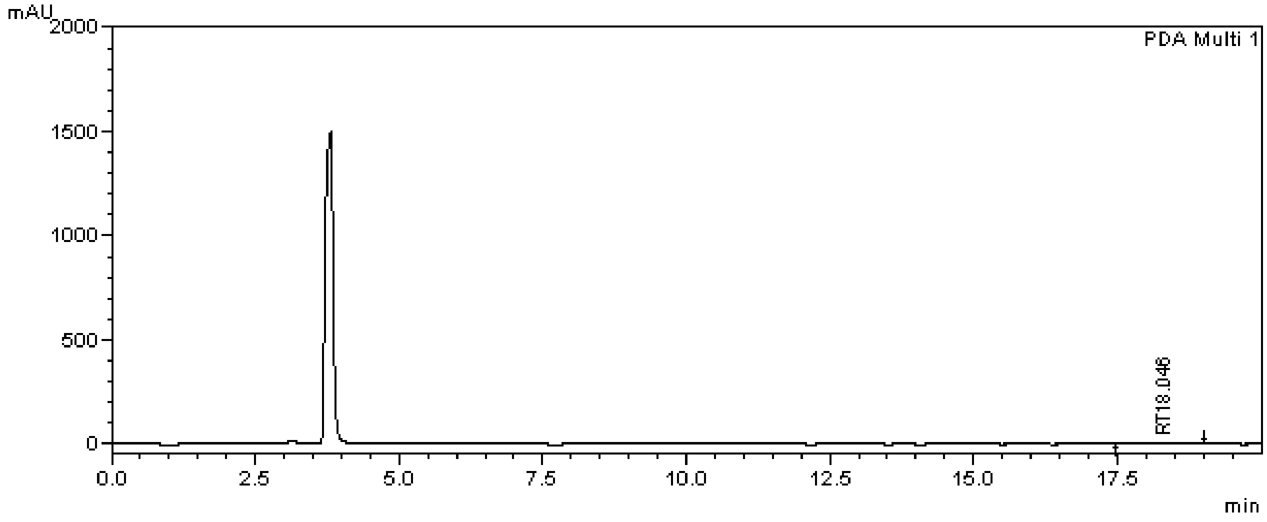

1 PDA Multi 1

<Results>

PDA Ch1 215nm 4nm

Peak#	Ret. Time	Area	Height	Area %	Height %	Resolution
1	18.046	299447	6364	100.000	100.000	0.000
Total		299447	6364	100.000	100.000	

Figure S8: Purity chromatogram of the **BrPQ8**

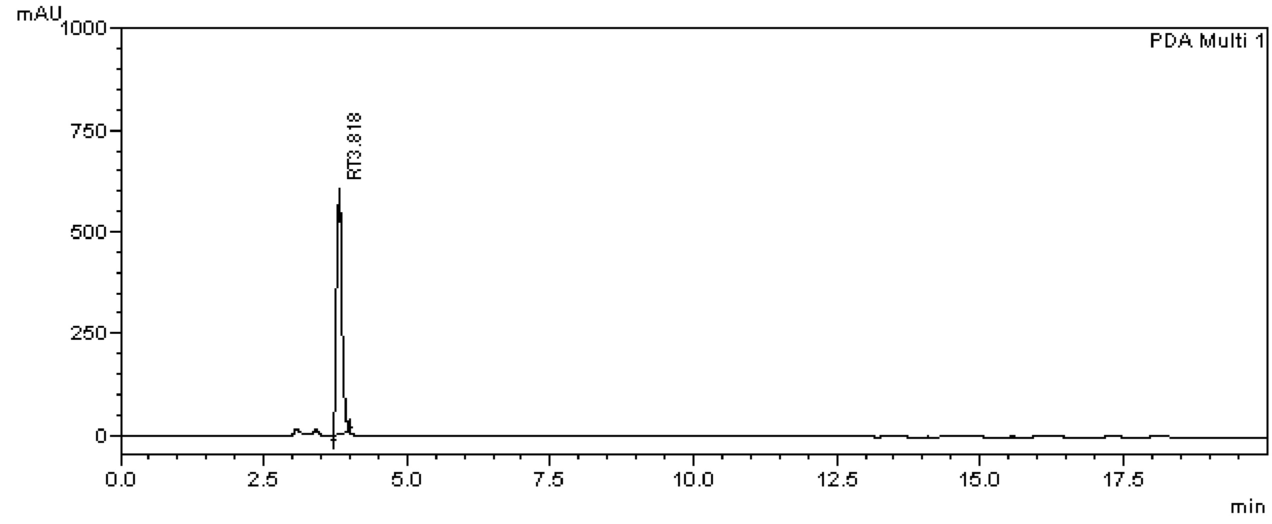

1 PDA Multi 1

<Results>

PDA Ch1 215nm 4nm

Peak#	Ret. Time	Area	Height	Area %	Height %	Resolution
1	3.818	4053535	602993	100.000	100.000	0.000
Total		4053535	602993	100.000	100.000	

Figure S9: Purity chromatogram of the **BrPQ9**

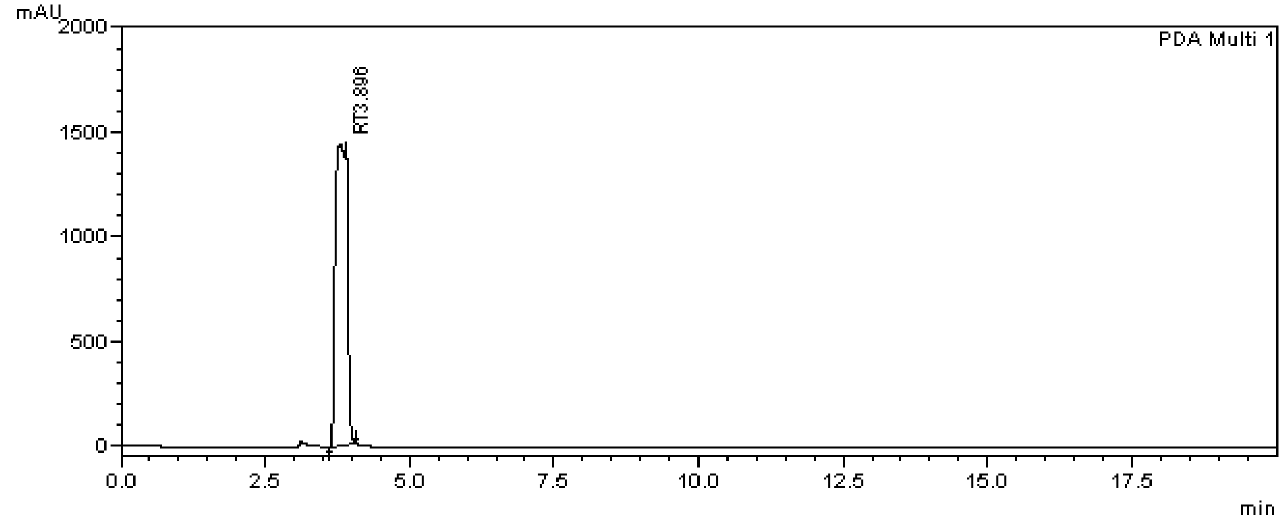

1 PDA Multi 1

<Results>

PDA Ch1 215nm 4nm

Peak#	Ret. Time	Area	Height	Area %	Height %	Resolution
1	3.896	21226698	1436539	100.000	100.000	0.000
Total		21226698	1436539	100.000	100.000	

Figure S10: Purity chromatogram of the **BrPQ10**

Developmental Therapeutics Program One Dose Mean Graph	NSC: D-825199 / 1	Conc: 1.00E-5 Molar	Test Date: Sep 28, 2020
	Experiment ID: 2009OS51		Report Date: May 06, 2021

Panel/Cell Line	Growth Percent
**Leukemia**	
CCRF-CEM	97.08
HL-60(TB)	92.53
K-562	97.93
MOLT-4	102.07
SR	140.54
**Non-Small Cell Lung Cancer**	
A549/ATCC	100.23
EKVX	99.19
HOP-62	99.17
HOP-92	88.49
NCI-H226	94.22
NCI-H23	97.75
NCI-H322M	100.65
NCI-H460	103.89
NCI-H522	90.88
**Colon Cancer**	
COLO 205	115.22
HCC-2998	102.29
HCT-116	101.35
HCT-15	105.05
HT29	107.79
KM12	104.86
SW-620	98.15
**CNS Cancer**	
SF-268	95.45
SF-295	104.26
SF-539	92.35
SNB-19	94.64
SNB-75	84.08
U251	97.50
**Melanoma**	
LOX IMVI	96.76
MALME-3M	93.61
M14	104.51
SK-MEL-2	97.06
SK-MEL-28	106.58
SK-MEL-5	99.04
UACC-257	94.00
UACC-62	92.11
**Ovarian Cancer**	
IGROV1	105.77
OVCAR-3	110.68
OVCAR-4	91.10
OVCAR-5	99.20
OVCAR-8	98.41
NCI/ADR-RES	99.05
SK-OV-3	103.83
**Renal Cancer**	
786-0	99.03
A498	95.23
ACHN	97.52
CAKI-1	100.57
RXF 393	102.99
SN12C	96.61
TK-10	99.87
UO-31	84.86
**Prostate Cancer**	
PC-3	98.83
DU-145	102.30
**Breast Cancer**	
MCF7	94.08
MDA-MB-231/ATCC	90.37
HS 578T	107.78
BT-549	99.14
T-47D	100.35
MDA-MB-468	93.42
Mean	99.35
Delta	15.27
Range	56.46

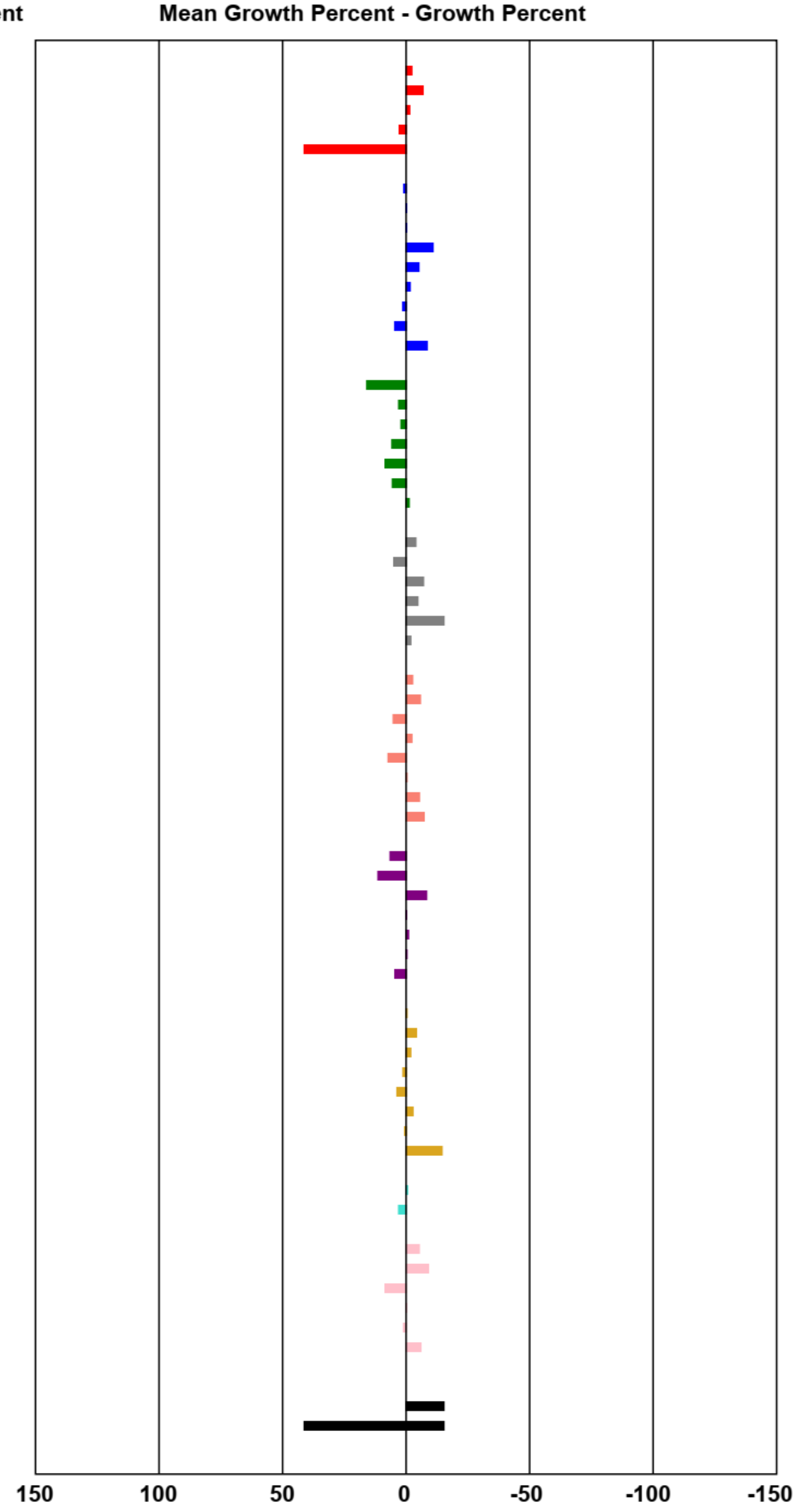

Figure S11: Single-dose *in vitro* antiproliferative activity of **BrPQ1**

Developmental Therapeutics Program

One Dose Mean Graph

NSC: D-827603 / 1 | **Conc:** 1.00E-5 Molar | **Test Date:** Jan 11, 2021

Experiment ID: 2101OS77 | **Report Date:** May 06, 2021

Panel/Cell Line	Growth Percent
Leukemia	
CCRF-CEM	16.54
HL-60(TB)	37.02
K-562	2.13
MOLT-4	-29.93
RPMI-8226	35.81
SR	69.34
Non-Small Cell Lung Cancer	
A549/ATCC	101.06
EKVX	68.75
HOP-62	90.73
HOP-92	-35.50
NCI-H226	92.19
NCI-H23	85.11
NCI-H322M	98.44
NCI-H460	102.81
NCI-H522	94.63
Colon Cancer	
COLO 205	125.55
HCC-2998	110.95
HCT-116	76.87
HCT-15	98.41
HT29	104.88
KM12	104.77
SW-620	106.73
CNS Cancer	
SF-268	100.43
SF-295	99.96
SF-539	98.18
SNB-19	101.11
SNB-75	67.72
U251	101.45
Melanoma	
LOX IMVI	58.08
MALME-3M	116.60
M14	103.63
MDA-MB-435	109.34
SK-MEL-2	116.86
SK-MEL-28	114.90
SK-MEL-5	90.54
UACC-257	84.78
UACC-62	94.44
Ovarian Cancer	
IGROV1	84.47
OVCAR-3	109.65
OVCAR-4	69.26
OVCAR-5	128.25
OVCAR-8	95.67
NCI/ADR-RES	98.12
SK-OV-3	85.70
Renal Cancer	
786-0	107.09
A498	95.46
ACHN	95.67
CAKI-1	99.31
RXF 393	105.92
SN12C	96.92
TK-10	151.82
UO-31	80.15
Prostate Cancer	
PC-3	78.55
DU-145	104.36
Breast Cancer	
MCF7	75.65
MDA-MB-231/ATCC	67.79
HS 578T	96.10
BT-549	106.19
T-47D	4.47
MDA-MB-468	-43.13
Mean	83.48
Delta	126.61
Range	194.95

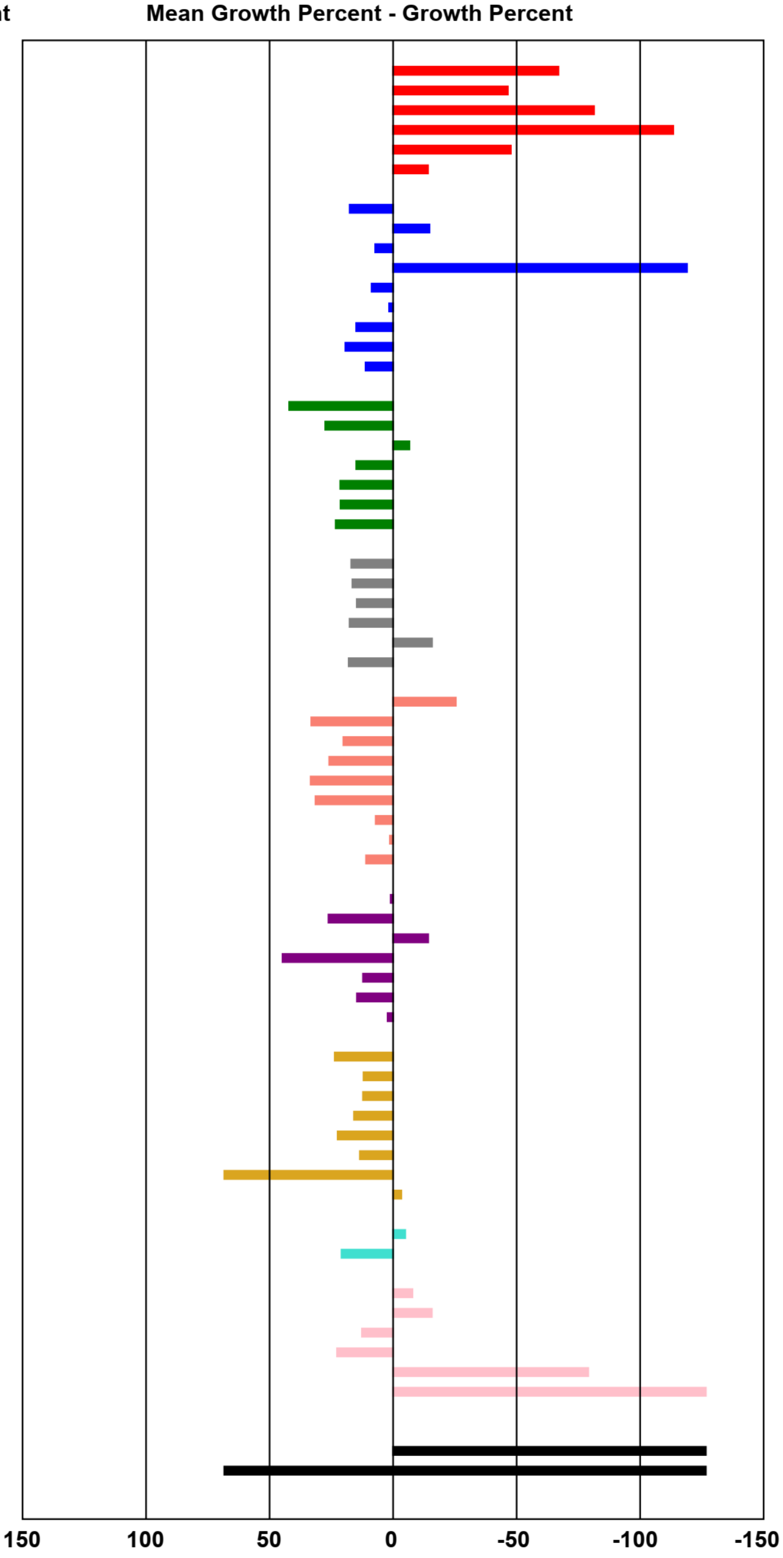

Figure S12: Single-dose *in vitro* antiproliferative activity of **BrPQ2**

Developmental Therapeutics Program
One Dose Mean Graph

**NSC:** D-827604 / 1	**Conc:** 1.00E-5 Molar	**Test Date:** Jan 11, 2021
**Experiment ID:** 2101OS77		**Report Date:** May 06, 2021

Panel/Cell Line	Growth Percent
**Leukemia**	
CCRF-CEM	17.87
HL-60(TB)	68.63
K-562	-21.33
MOLT-4	-30.61
RPMI-8226	55.16
SR	43.60
**Non-Small Cell Lung Cancer**	
A549/ATCC	100.15
EKVX	79.30
HOP-62	83.24
HOP-92	-35.80
NCI-H226	86.51
NCI-H23	87.72
NCI-H322M	103.92
NCI-H460	101.58
NCI-H522	96.10
**Colon Cancer**	
COLO 205	124.38
HCC-2998	104.40
HCT-116	35.36
HCT-15	95.95
HT29	108.29
KM12	103.64
SW-620	102.59
**CNS Cancer**	
SF-268	97.31
SF-295	98.08
SF-539	99.99
SNB-19	100.87
SNB-75	88.99
U251	103.63
**Melanoma**	
LOX IMVI	29.86
MALME-3M	111.88
M14	106.36
MDA-MB-435	103.47
SK-MEL-2	111.13
SK-MEL-28	112.42
SK-MEL-5	94.00
UACC-257	93.82
UACC-62	92.23
**Ovarian Cancer**	
IGROV1	94.46
OVCAR-3	104.72
OVCAR-4	75.61
OVCAR-5	108.16
OVCAR-8	92.73
NCI/ADR-RES	95.75
SK-OV-3	85.55
**Renal Cancer**	
786-0	106.06
A498	97.23
ACHN	101.56
CAKI-1	106.07
RXF 393	97.65
SN12C	98.87
TK-10	145.33
UO-31	87.51
**Prostate Cancer**	
PC-3	87.78
DU-145	106.07
**Breast Cancer**	
MCF7	76.01
MDA-MB-231/ATCC	70.05
HS 578T	96.71
BT-549	104.50
T-47D	15.71
MDA-MB-468	-4.77
Mean	83.40
Delta	119.20
Range	181.13

Mean Growth Percent - Growth Percent

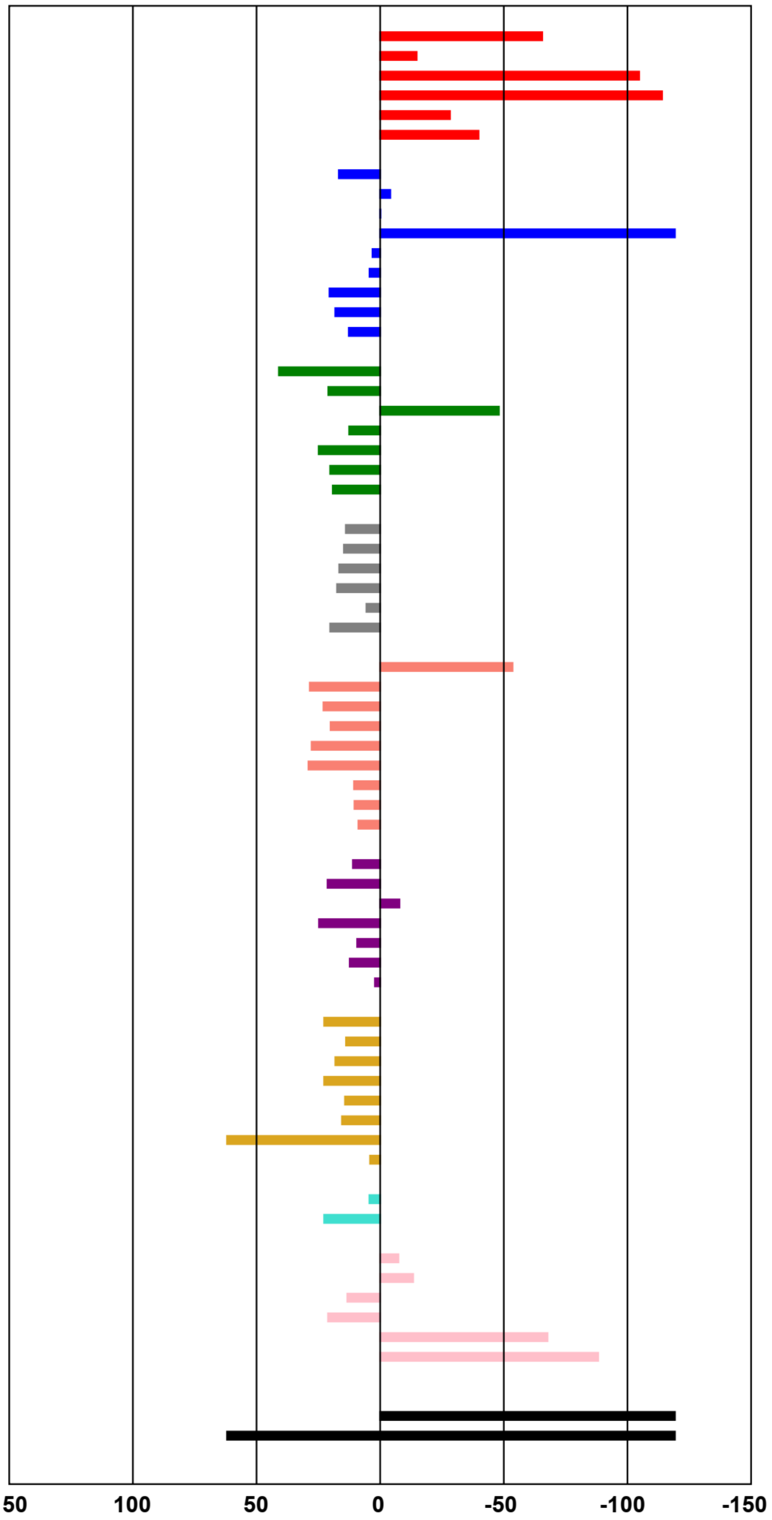

Figure S13: Single-dose *in vitro* antiproliferative activity of **BrPQ3**

Developmental Therapeutics Program

One Dose Mean Graph

**NSC:** D-827602 / 1	**Conc:** 1.00E-5 Molar
**Test Date:** Jan 11, 2021	**Experiment ID:** 2101OS77
**Report Date:** May 06, 2021	

Panel/Cell Line	Growth Percent
Leukemia	
CCRF-CEM	-0.58
HL-60(TB)	54.71
K-562	-17.91
MOLT-4	-6.02
RPMI-8226	-10.84
SR	26.12
Non-Small Cell Lung Cancer	
A549/ATCC	94.37
EKVX	72.86
HOP-62	81.13
HOP-92	25.03
NCI-H226	83.23
NCI-H23	88.83
NCI-H322M	100.43
NCI-H460	96.36
NCI-H522	29.20
Colon Cancer	
COLO 205	115.29
HCC-2998	108.03
HCT-116	20.01
HCT-15	82.69
HT29	106.31
KM12	90.74
SW-620	48.66
CNS Cancer	
SF-268	97.69
SF-295	106.15
SF-539	96.01
SNB-19	91.10
SNB-75	62.24
U251	87.41
Melanoma	
LOX IMVI	6.64
MALME-3M	101.24
M14	99.01
MDA-MB-435	103.43
SK-MEL-2	103.82
SK-MEL-28	104.65
SK-MEL-5	81.35
UACC-257	88.14
UACC-62	80.82
Ovarian Cancer	
IGROV1	74.80
OVCAR-3	103.26
OVCAR-4	81.40
OVCAR-5	127.60
OVCAR-8	96.98
NCI/ADR-RES	102.81
SK-OV-3	85.98
Renal Cancer	
786-0	96.99
A498	86.43
ACHN	72.29
CAKI-1	94.28
RXF 393	104.51
SN12C	97.10
TK-10	157.37
UO-31	79.15
Prostate Cancer	
PC-3	71.01
DU-145	104.95
Breast Cancer	
MCF7	52.33
MDA-MB-231/ATCC	69.17
HS 578T	99.37
BT-549	110.26
T-47D	17.58
MDA-MB-468	-61.36
Mean	75.38
Delta	136.74
Range	218.73

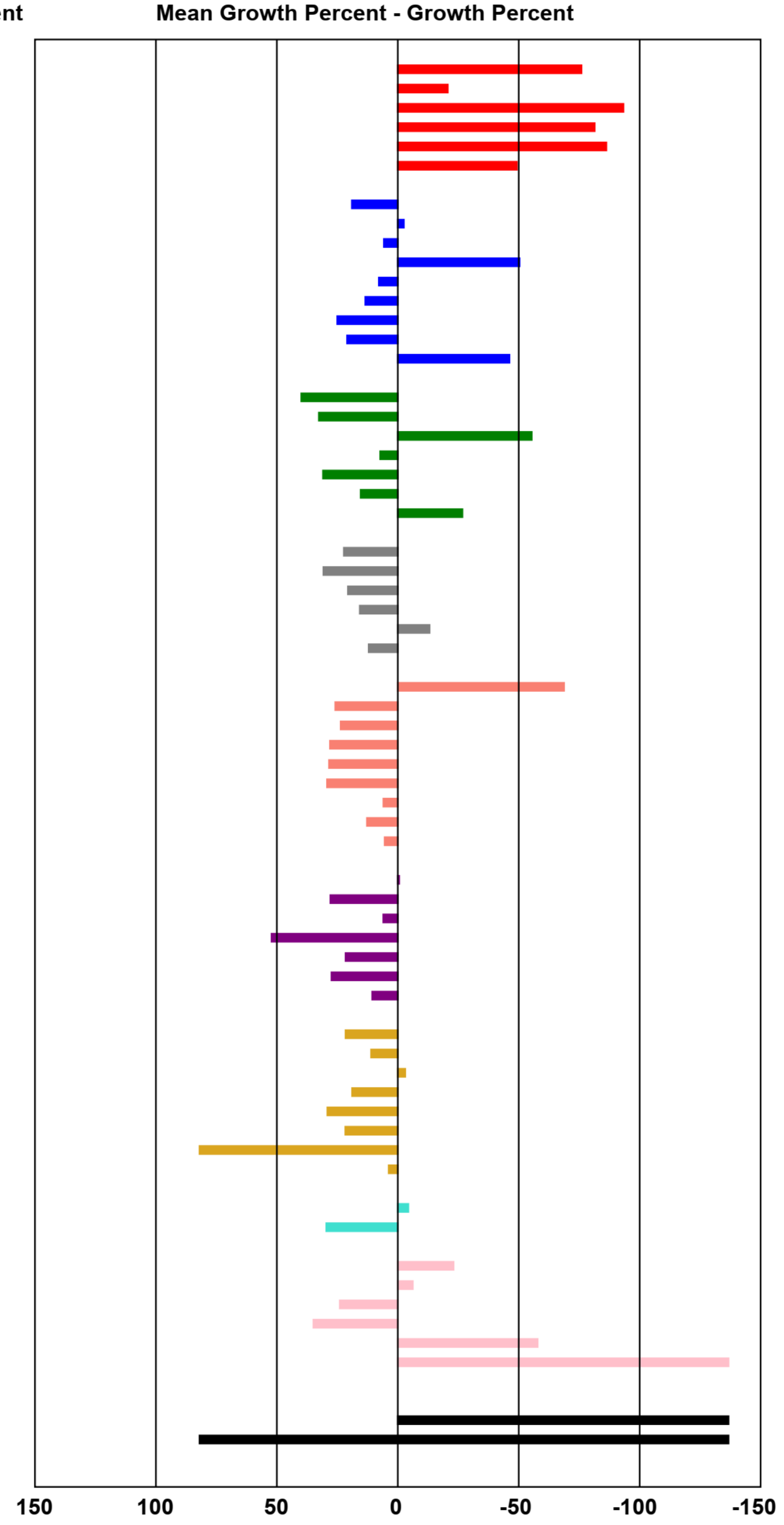

Figure S14: Single-dose *in vitro* antiproliferative activity of **BrPQ4**

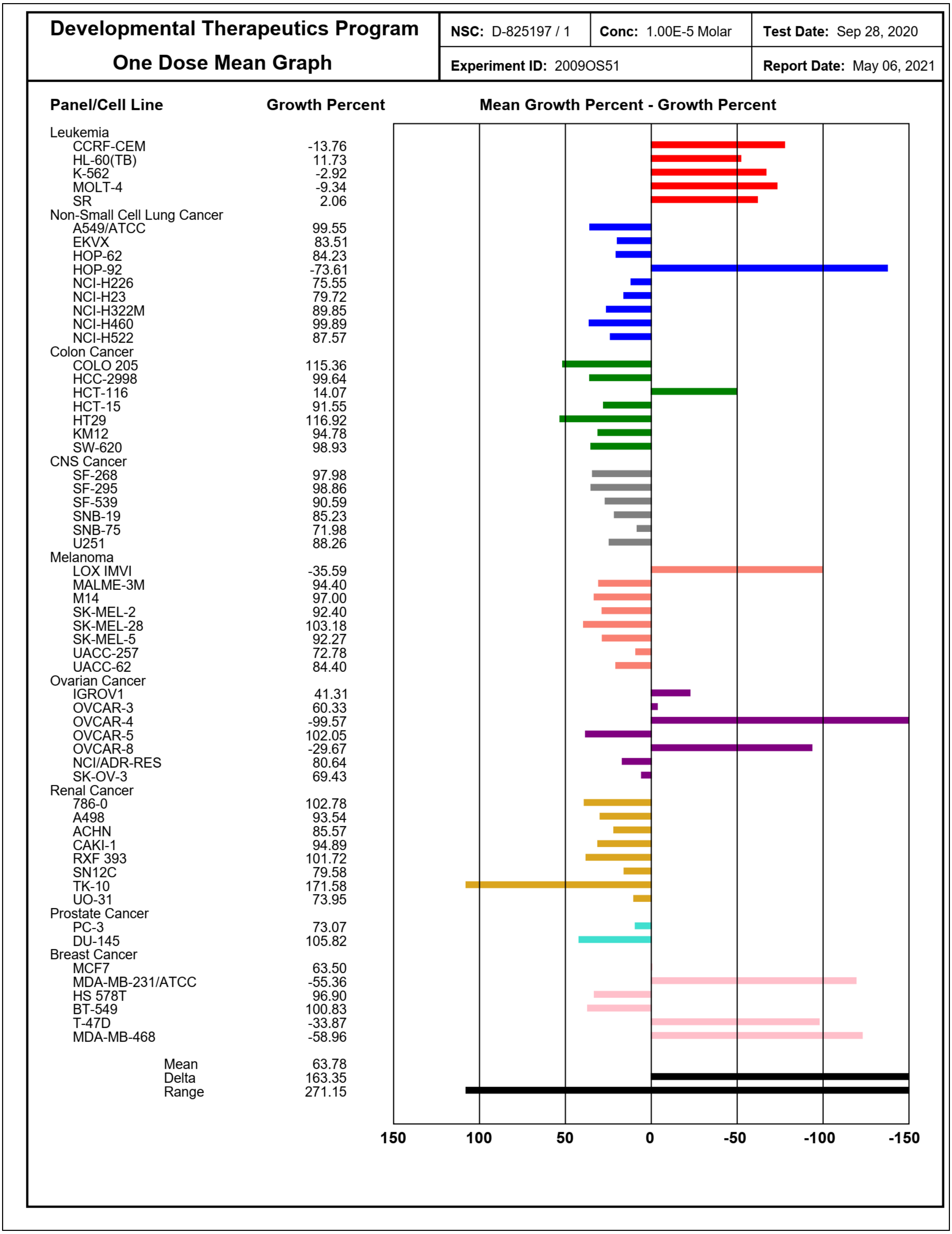

Figure S15: Single-dose *in vitro* antiproliferative activity of **BrPQ5**

Developmental Therapeutics Program	NSC: D-825198 / 1	Conc: 1.00E-5 Molar	Test Date: Sep 28, 2020
One Dose Mean Graph	Experiment ID: 2009OS51		Report Date: May 06, 2021

Panel/Cell Line	Growth Percent
**Leukemia**	
CCRF-CEM	3.35
HL-60(TB)	-12.49
K-562	0.43
MOLT-4	2.57
SR	74.75
**Non-Small Cell Lung Cancer**	
A549/ATCC	103.07
EKVX	92.37
HOP-62	83.65
HOP-92	-8.14
NCI-H226	72.04
NCI-H23	67.51
NCI-H322M	96.23
NCI-H460	99.98
NCI-H522	80.54
**Colon Cancer**	
COLO 205	122.61
HCC-2998	118.60
HCT-116	51.98
HCT-15	98.61
HT29	106.65
KM12	105.87
SW-620	104.98
**CNS Cancer**	
SF-268	94.08
SF-295	100.56
SF-539	94.94
SNB-19	93.99
SNB-75	56.29
U251	97.34
**Melanoma**	
LOX IMVI	25.98
MALME-3M	91.44
M14	87.11
SK-MEL-2	95.50
SK-MEL-28	104.19
SK-MEL-5	89.04
UACC-257	68.04
UACC-62	81.53
**Ovarian Cancer**	
IGROV1	58.61
OVCAR-3	87.56
OVCAR-4	56.74
OVCAR-5	95.70
OVCAR-8	91.50
NCI/ADR-RES	96.53
SK-OV-3	87.96
**Renal Cancer**	
786-0	103.50
A498	108.94
ACHN	85.82
CAKI-1	96.00
RXF 393	113.47
SN12C	84.96
TK-10	155.05
UO-31	71.72
**Prostate Cancer**	
PC-3	73.78
DU-145	104.37
**Breast Cancer**	
MCF7	18.43
MDA-MB-231/ATCC	34.26
HS 578T	91.24
BT-549	96.44
T-47D	-35.26
MDA-MB-468	-65.71
Mean	75.12
Delta	140.83
Range	220.76

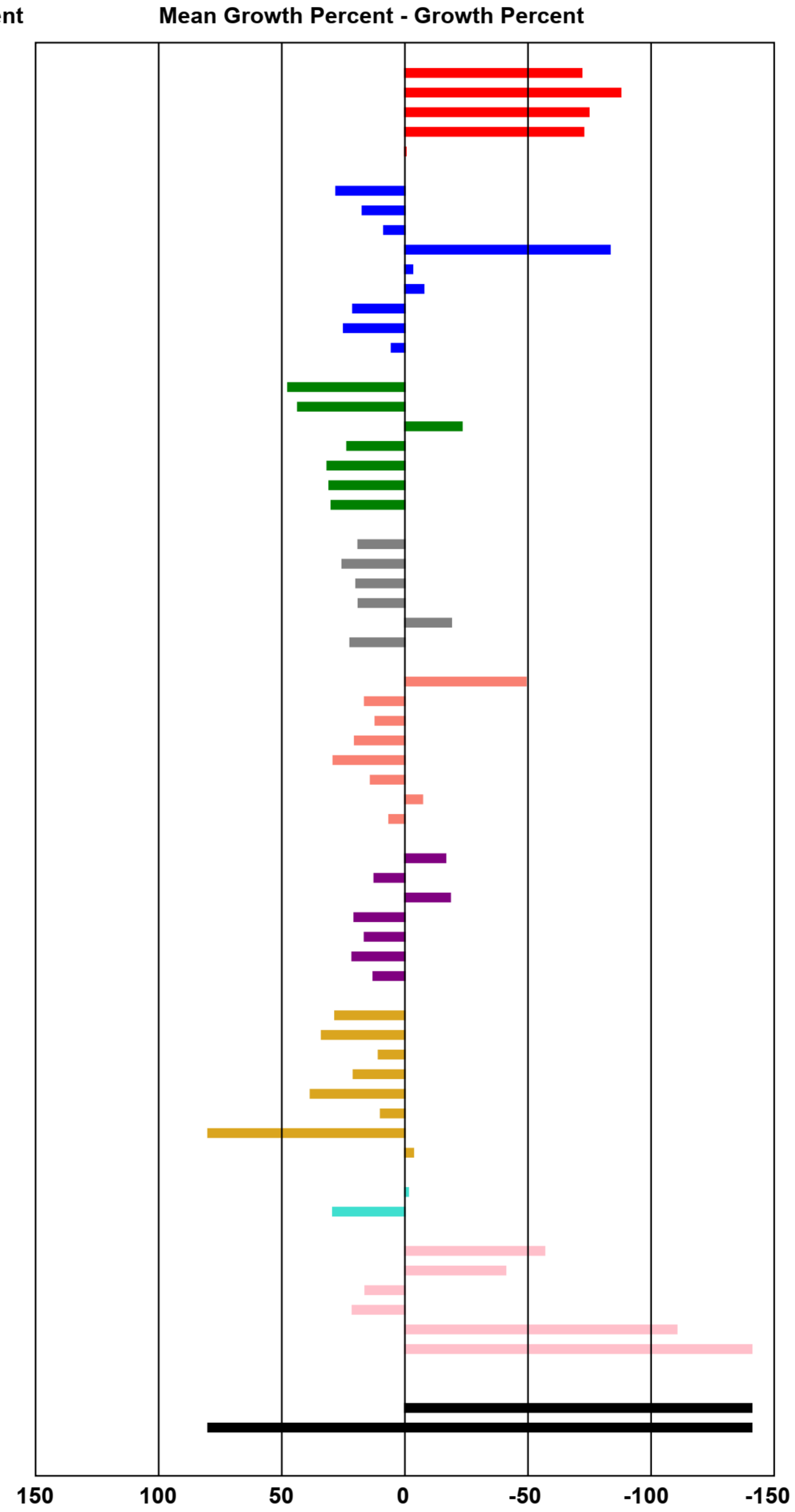

Figure S16: Single-dose *in vitro* antiproliferative activity of **BrPQ6**

Developmental Therapeutics Program

One Dose Mean Graph

**NSC:** D-827608 / 1	**Conc:** 1.00E-5 Molar
**Test Date:** Jan 11, 2021	**Experiment ID:** 2101OS77
**Report Date:** May 06, 2021	

Panel/Cell Line	Growth Percent
Leukemia	
CCRF-CEM	-16.76
HL-60(TB)	-37.74
K-562	-2.41
MOLT-4	-14.71
RPMI-8226	29.84
SR	43.62
Non-Small Cell Lung Cancer	
A549/ATCC	102.80
EKVX	34.16
HOP-62	104.16
HOP-92	3.51
NCI-H226	89.58
NCI-H23	76.87
NCI-H322M	102.02
NCI-H460	99.58
NCI-H522	97.24
Colon Cancer	
COLO 205	115.55
HCC-2998	96.37
HCT-116	34.73
HCT-15	92.34
HT29	106.91
KM12	99.85
SW-620	102.94
CNS Cancer	
SF-268	98.35
SF-295	92.91
SF-539	98.89
SNB-19	97.38
SNB-75	73.19
U251	101.60
Melanoma	
LOX IMVI	-54.58
MALME-3M	114.42
M14	96.47
MDA-MB-435	103.07
SK-MEL-2	111.67
SK-MEL-28	110.55
SK-MEL-5	89.09
UACC-257	98.52
UACC-62	96.42
Ovarian Cancer	
IGROV1	90.88
OVCAR-3	108.54
OVCAR-4	55.12
OVCAR-5	111.27
OVCAR-8	100.21
NCI/ADR-RES	82.71
SK-OV-3	99.96
Renal Cancer	
786-0	105.19
A498	91.12
ACHN	101.97
CAKI-1	93.97
RXF 393	87.85
SN12C	98.72
TK-10	156.64
UO-31	90.79
Prostate Cancer	
PC-3	85.01
DU-145	108.56
Breast Cancer	
MCF7	64.15
MDA-MB-231/ATCC	65.07
HS 578T	97.37
BT-549	100.28
T-47D	41.59
MDA-MB-468	1.26
Mean	78.78
Delta	133.36
Range	211.22

Mean Growth Percent - Growth Percent

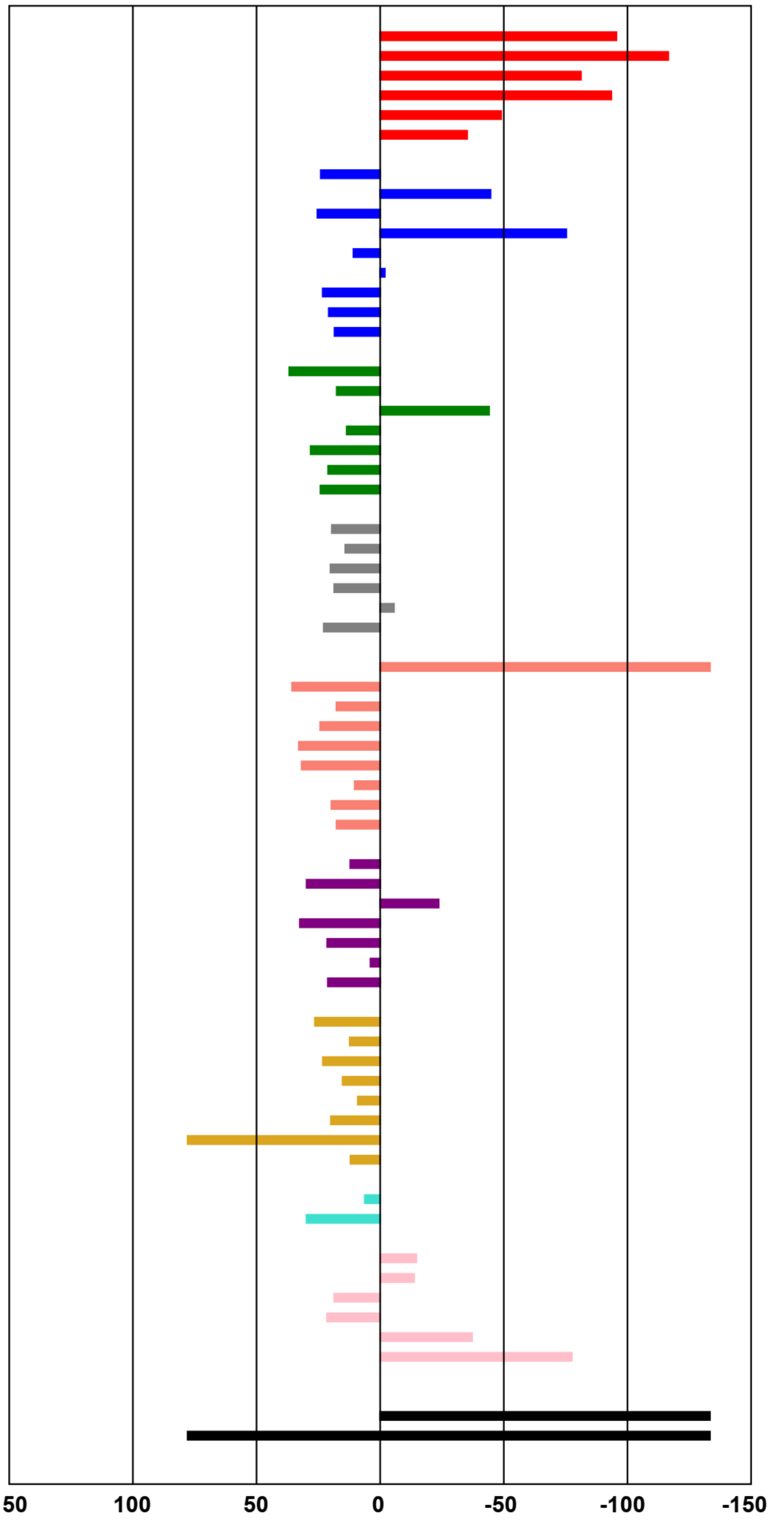

Figure S17: Single-dose *in vitro* antiproliferative activity of **BrPQ7**

Developmental Therapeutics Program
One Dose Mean Graph

NSC: D-827605 / 1	Conc: 1.00E-5 Molar	Test Date: Jan 11, 2021
Experiment ID: 2101OS77		Report Date: May 06, 2021

Panel/Cell Line	Growth Percent
**Leukemia**	
CCRF-CEM	-27.95
HL-60(TB)	-34.64
K-562	-7.89
MOLT-4	-41.56
RPMI-8226	70.79
SR	71.24
**Non-Small Cell Lung Cancer**	
A549/ATCC	98.74
EKVX	80.82
HOP-62	104.26
HOP-92	-60.81
NCI-H226	88.25
NCI-H23	89.82
NCI-H322M	105.77
NCI-H460	101.10
NCI-H522	89.56
**Colon Cancer**	
COLO 205	125.26
HCC-2998	102.39
HCT-116	90.75
HCT-15	97.90
HT29	110.62
KM12	59.21
SW-620	107.07
**CNS Cancer**	
SF-268	95.27
SF-295	94.38
SF-539	101.16
SNB-19	84.87
SNB-75	77.11
U251	83.97
**Melanoma**	
LOX IMVI	73.67
MALME-3M	116.58
M14	104.49
MDA-MB-435	101.58
SK-MEL-2	106.07
SK-MEL-28	112.10
SK-MEL-5	69.73
UACC-257	95.96
UACC-62	87.99
**Ovarian Cancer**	
IGROV1	86.03
OVCAR-3	106.89
OVCAR-4	14.06
OVCAR-5	120.80
OVCAR-8	96.02
NCI/ADR-RES	97.26
SK-OV-3	83.75
**Renal Cancer**	
786-0	106.36
A498	95.01
ACHN	99.67
CAKI-1	99.12
RXF 393	104.10
SN12C	100.38
TK-10	165.14
UO-31	83.55
**Prostate Cancer**	
PC-3	69.66
DU-145	109.06
**Breast Cancer**	
MCF7	72.14
MDA-MB-231/ATCC	33.35
HS 578T	97.61
BT-549	112.25
T-47D	-8.45
MDA-MB-468	-66.65
Mean	78.38
Delta	145.03
Range	231.79

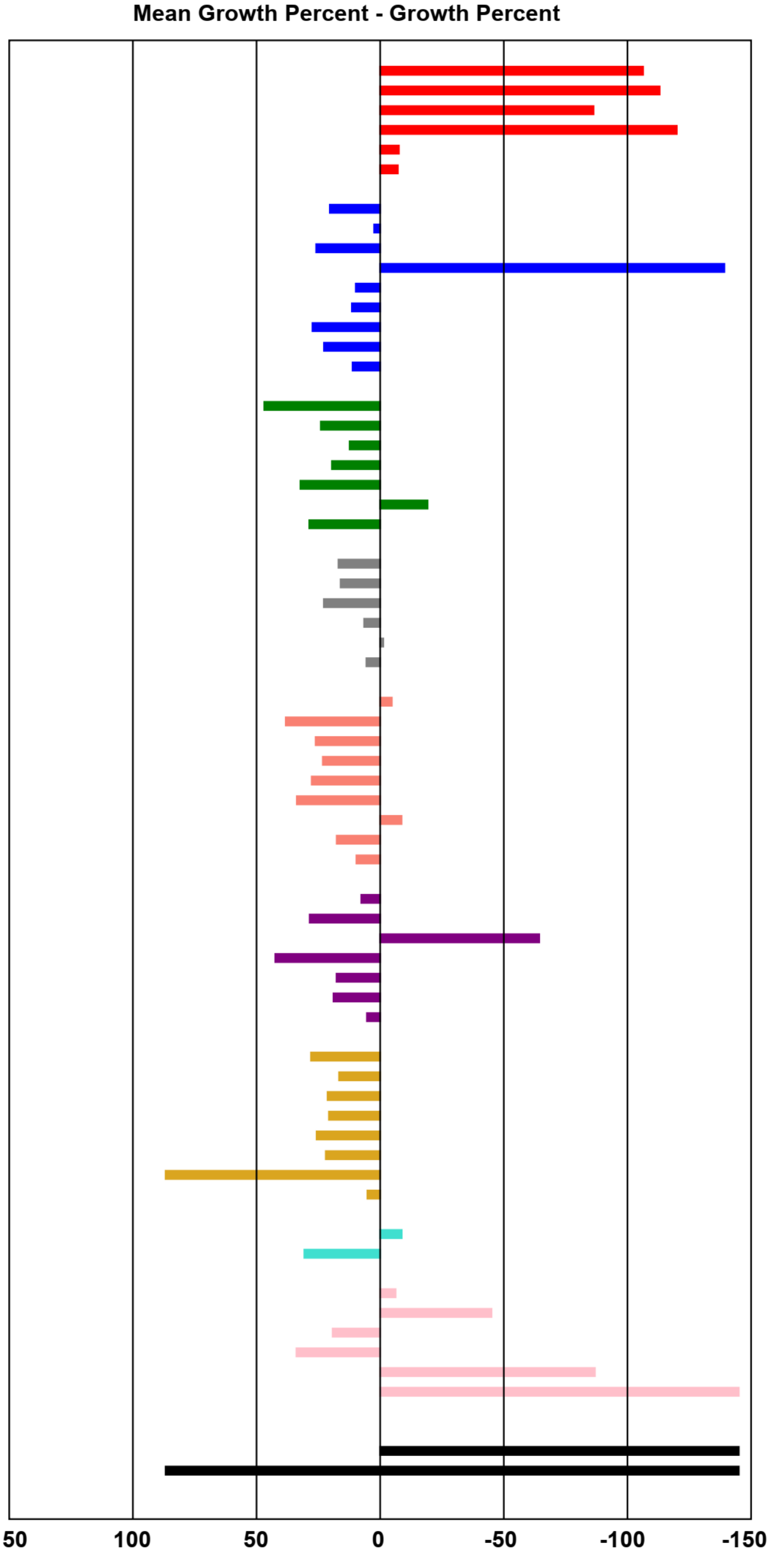

Figure S18: Single-dose *in vitro* antiproliferative activity of **BrPQ8**

**Developmental Therapeutics Program**	**NSC:** D-827606 / 1	**Conc:** 1.00E-5 Molar	**Test Date:** Jan 11, 2021
**One Dose Mean Graph**	**Experiment ID:** 2101OS77		**Report Date:** May 06, 2021

Panel/Cell Line	Growth Percent
Leukemia	
CCRF-CEM	11.57
HL-60(TB)	44.23
K-562	3.43
MOLT-4	-10.20
RPMI-8226	71.01
SR	56.86
Non-Small Cell Lung Cancer	
A549/ATCC	101.81
EKVX	76.34
HOP-62	92.92
HOP-92	-22.95
NCI-H226	92.96
NCI-H23	90.04
NCI-H322M	96.50
NCI-H460	100.06
NCI-H522	96.47
Colon Cancer	
COLO 205	121.86
HCC-2998	105.46
HCT-116	85.58
HCT-15	100.83
HT29	109.41
KM12	97.37
SW-620	107.18
CNS Cancer	
SF-268	96.65
SF-295	96.75
SF-539	106.16
SNB-19	93.85
SNB-75	74.29
U251	102.69
Melanoma	
LOX IMVI	77.20
MALME-3M	112.45
M14	105.16
MDA-MB-435	103.06
SK-MEL-2	113.75
SK-MEL-28	110.46
SK-MEL-5	90.22
UACC-257	94.30
UACC-62	87.38
Ovarian Cancer	
IGROV1	90.94
OVCAR-3	104.29
OVCAR-4	60.30
OVCAR-5	115.82
OVCAR-8	98.34
NCI/ADR-RES	97.36
SK-OV-3	87.65
Renal Cancer	
786-0	103.45
A498	95.19
ACHN	100.47
CAKI-1	95.63
RXF 393	104.54
SN12C	102.19
TK-10	149.99
UO-31	88.12
Prostate Cancer	
PC-3	86.01
DU-145	109.49
Breast Cancer	
MCF7	77.50
MDA-MB-231/ATCC	69.39
HS 578T	99.68
BT-549	107.41
T-47D	30.30
MDA-MB-468	36.26
Mean	86.72
Delta	109.67
Range	172.94

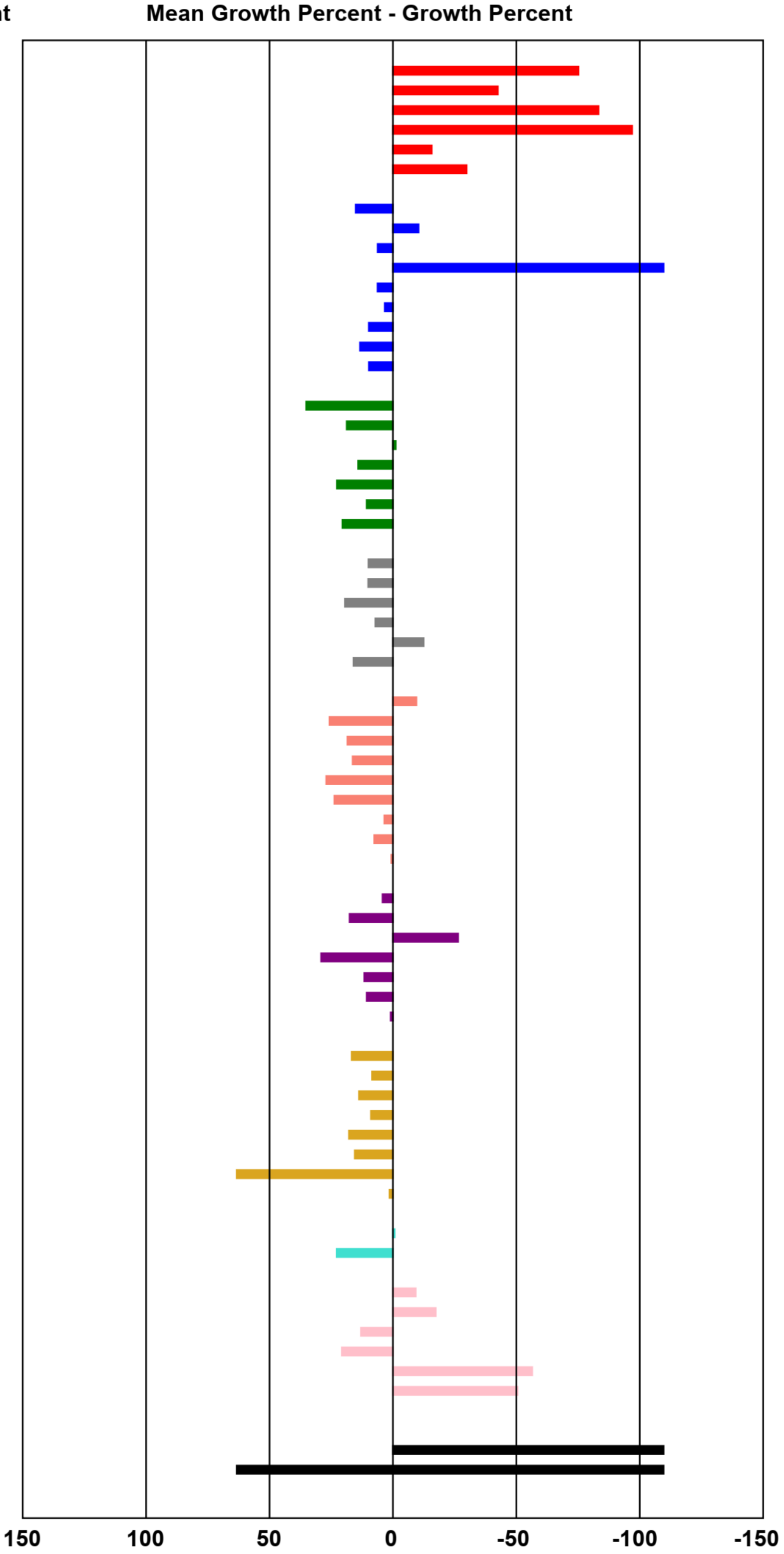

Figure S19: Single-dose *in vitro* antiproliferative activity of **BrPQ9**

Developmental Therapeutics Program One Dose Mean Graph	NSC: D-827607 / 1	Conc: 1.00E-5 Molar	Test Date: Jan 11, 2021
	Experiment ID: 2101OS77		Report Date: May 06, 2021

Panel/Cell Line	Growth Percent
Leukemia	
CCRF-CEM	13.17
HL-60(TB)	-44.23
K-562	4.46
MOLT-4	-7.79
RPMI-8226	100.34
SR	77.65
Non-Small Cell Lung Cancer	
A549/ATCC	107.42
EKVX	73.73
HOP-62	97.62
HOP-92	68.20
NCI-H226	85.57
NCI-H23	85.23
NCI-H322M	102.22
NCI-H460	101.88
NCI-H522	96.94
Colon Cancer	
COLO 205	125.78
HCC-2998	101.88
HCT-116	90.62
HCT-15	98.08
HT29	107.81
KM12	93.67
SW-620	106.53
CNS Cancer	
SF-268	97.83
SF-295	94.68
SF-539	105.01
SNB-19	98.45
SNB-75	83.71
U251	102.58
Melanoma	
LOX IMVI	20.33
MALME-3M	121.90
M14	105.68
MDA-MB-435	101.63
SK-MEL-2	113.53
SK-MEL-28	107.25
SK-MEL-5	89.10
UACC-257	102.45
UACC-62	92.06
Ovarian Cancer	
IGROV1	102.46
OVCAR-3	108.81
OVCAR-4	66.82
OVCAR-5	107.62
OVCAR-8	100.09
NCI/ADR-RES	97.26
SK-OV-3	93.94
Renal Cancer	
786-0	105.32
A498	102.79
ACHN	101.07
CAKI-1	101.65
RXF 393	100.35
SN12C	99.69
TK-10	135.01
UO-31	95.83
Prostate Cancer	
PC-3	85.97
DU-145	107.49
Breast Cancer	
MCF7	78.24
MDA-MB-231/ATCC	88.28
HS 578T	97.27
BT-549	100.66
T-47D	80.52
MDA-MB-468	28.14
Mean	88.47
Delta	132.70
Range	179.24

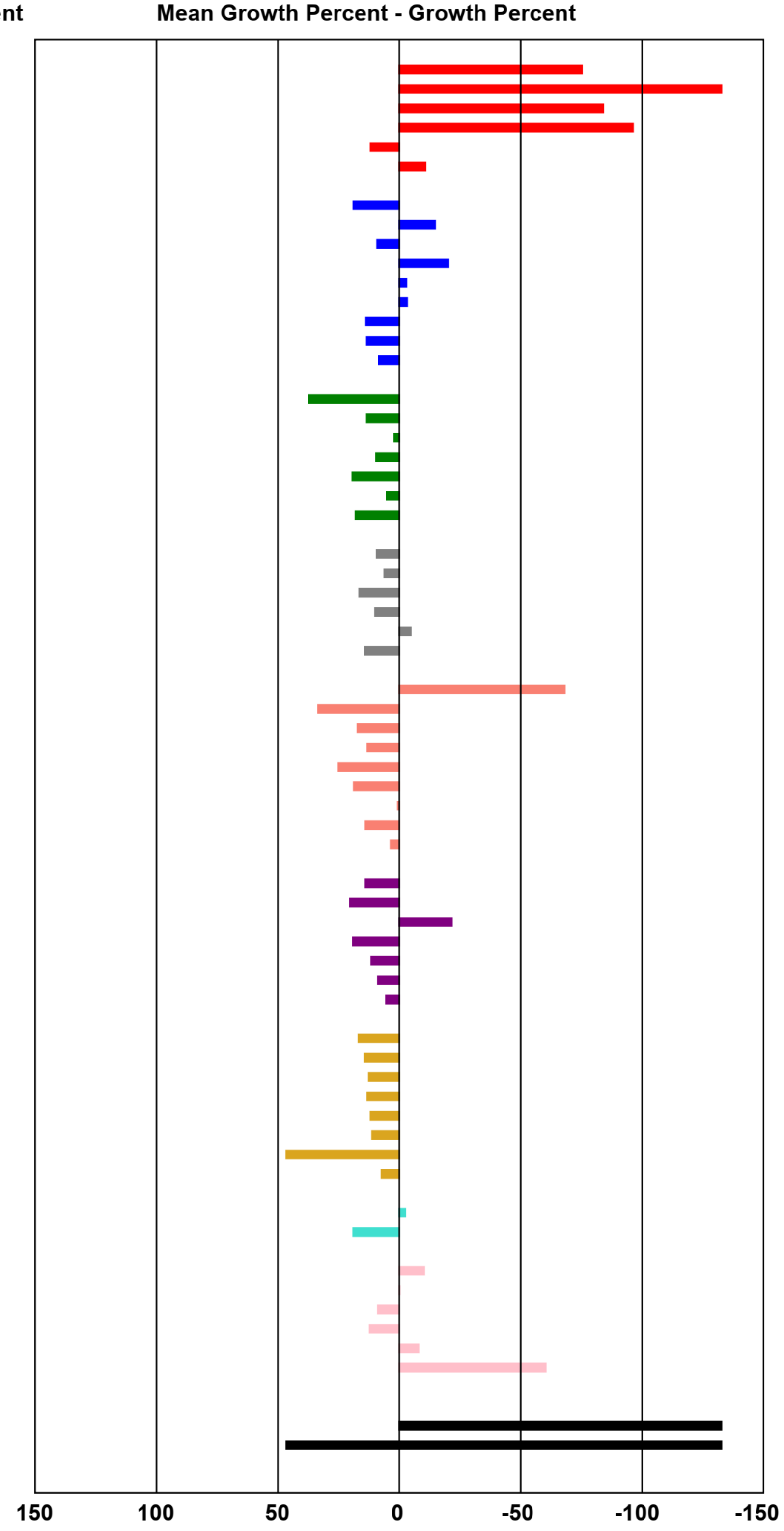

Figure S20: Single-dose *in vitro* antiproliferative activity of **BrPQ10**

Table S1. Antiproliferative activity data as per single dose assay at 10 µM concentration as percent cell growth of the selected hybrid molecules.

Molecules / Panel/Cancer Cell Line	Growth Percentage of Cell Lines in NCI 60									
	**BrPQ1**	**BrPQ2**	**BrPQ3**	**BrPQ4**	**BrPQ5**	**BrPQ6**	**BrPQ7**	**BrPQ8**	**BrPQ9**	**BrPQ10**
Mean Growth (%)	99.35	83.48	83.40	75.38	63.78	75.12	78.78	78.38	86.72	88.47
Standard Deviation (SD)	8.04	38.74	37.13	40.27	55.63	41.67	41.93	47.15	31.59	32.24
**Leukemia**										
CCRF-CEM	97.08	16.54	17.87	-0.58	-13.76	3.35	-16.76	-27.95	11.57	13.17
HL-60(TB)	92.53	37.02	68.63	54.71	11.73	-12.49	-37.74	-34.64	44.23	-44.23
K-562	97.93	2.13	-21.33	-17.91	-2.92	0.43	-2.41	-7.89	3.43	4.46
MOLT-4	102.07	-29.93	-30.61	-6.02	-9.34	2.57	-14.71	-41.56	-10.20	-7.79
RPMI-8226	ND*	35.81	55.16	-10.84	ND*	ND*	29.84	70.79	71.01	100.34
SR	140.54	69.34	43.60	26.12	2.06	74.75	43.62	71.24	56.86	77.65
**Non-Small Cell Lung Cancer**										
A549/ATCC	100.23	101.06	100.15	94.37	99.55	103.07	102.80	98.74	101.81	107.42
EKVX	99.19	68.75	79.30	72.86	83.51	92.37	34.16	80.82	76.34	73.73
HOP-62	99.17	90.73	83.24	81.13	84.23	83.65	104.16	104.26	92.92	97.62
HOP-92	88.49	-35.50	-35.80	25.03	-73.61	-8.14	3.51	-60.81	-22.95	68.20
NCI-H226	94.22	92.19	86.51	83.23	75.55	72.04	89.58	88.25	92.96	85.57
NCI-H23	97.75	85.11	87.72	88.83	79.72	67.51	76.87	89.82	90.04	85.23
NCI-H322M	100.65	98.44	103.92	100.43	89.85	96.23	102.02	105.77	96.50	102.22
NCI-H460	103.89	102.81	101.58	96.36	99.89	99.98	99.58	101.10	100.06	101.88
NCI-H522	90.88	94.63	96.10	29.20	87.57	80.54	97.24	89.56	96.47	96.94
**Colon Cancer**										
COLO 205	115.22	125.55	124.38	115.29	115.36	122.61	115.55	125.26	121.86	125.78

HCC-2998	102.29	110.95	104.40	108.03	99.64	118.60	96.37	102.39	105.46	101.88
HCT-116	101.35	76.87	35.36	20.01	14.07	51.98	34.73	90.75	85.58	90.62
HCT-15	105.05	98.41	95.95	82.69	91.55	98.61	92.34	97.90	100.83	98.08
HT29	107.79	104.88	108.29	106.31	116.92	106.65	106.91	110.62	109.41	107.81
KM12	104.86	104.77	103.64	90.74	94.78	105.87	99.85	59.21	97.37	93.67
SW-620	98.15	106.73	102.59	48.66	98.93	104.98	102.94	107.07	107.18	106.53
**CNS Cancer**										
SF-268	95.45	100.43	97.31	97.69	97.98	94.08	98.35	95.27	96.65	97.83
SF-295	104.26	99.96	98.08	106.15	98.86	100.56	92.91	94.38	96.75	94.68
SF-539	92.35	98.18	99.99	96.01	90.59	94.94	98.89	101.16	106.16	105.01
SNB-19	94.64	101.11	100.87	91.10	85.23	93.99	97.38	84.87	93.85	98.45
SNB-75	84.08	67.72	88.99	62.24	71.98	56.29	73.19	77.11	74.29	83.71
U251	97.50	101.45	103.63	87.41	88.26	97.34	101.60	83.97	102.69	102.58
**Melanoma**										
LOX IMVI	96.76	58.08	29.86	6.64	-35.59	25.98	-54.58	73.67	77.20	20.33
MALME-3M	93.61	116.60	111.88	101.24	94.40	91.44	114.42	116.58	112.45	121.90
M14	104.51	103.63	106.36	99.01	97.00	87.11	96.47	104.49	105.16	105.68
MDA-MB-435	ND*	109.34	103.47	103.43	ND*	ND*	103.07	101.58	103.06	101.63
SK-MEL-2	97.06	116.86	111.13	103.82	92.40	95.50	111.67	106.07	113.75	113.53
SK-MEL-28	106.58	114.90	112.42	104.65	103.18	104.19	110.55	112.10	110.46	107.25
SK-MEL-5	99.04	90.54	94.00	81.35	92.27	89.04	89.09	69.73	90.22	89.10
UACC-257	94.00	84.78	93.82	88.14	72.78	68.04	98.52	95.96	94.30	102.45
UACC-62	92.11	94.44	92.23	80.82	84.40	81.53	96.42	87.99	87.38	92.06
**Ovarian Cancer**										
IGROV1	105.77	84.47	94.46	74.80	41.31	58.61	90.88	86.03	90.94	102.46
OVCAR-3	110.68	109.65	104.72	103.26	60.33	87.56	108.54	106.89	104.29	108.81

OVCAR-4	91.10	69.26	75.61	81.40	-99.57	56.74	55.12	14.06	60.30	66.82
OVCAR-5	99.20	128.25	108.16	127.60	102.05	95.70	111.27	120.80	115.82	107.62
OVCAR-8	98.41	95.67	92.73	96.98	-29.67	91.50	100.21	96.02	98.34	100.09
NCI/ADR-RES	99.05	98.12	95.75	102.81	80.64	96.53	82.71	97.26	97.36	97.26
SK-OV-3	103.83	85.70	85.55	85.98	69.43	87.96	99.96	83.75	87.65	93.94
**Renal Cancer**										
786-0	99.03	107.09	106.06	96.99	102.78	103.50	105.19	106.36	103.45	105.32
A498	95.23	95.46	97.23	86.43	93.54	108.94	91.12	95.01	95.19	102.79
ACHN	97.52	95.67	101.56	72.29	85.57	85.82	101.97	99.67	100.47	101.07
CAKI-1	100.57	99.31	106.07	94.28	94.89	96.00	93.97	99.12	95.63	101.65
RXF 393	102.99	105.92	97.65	104.51	101.72	113.47	87.85	104.10	104.54	100.35
SN12C	96.61	96.92	98.87	97.10	79.58	84.96	98.72	100.38	102.19	99.69
TK-10	99.87	151.82	145.33	157.37	171.58	155.05	156.64	165.14	149.99	135.01
UO-31	84.86	80.15	87.51	79.15	73.95	71.72	90.79	83.55	88.12	95.83
**Prostate Cancer**										
PC-3	98.83	78.55	87.78	71.01	73.07	73.78	85.01	69.66	86.01	85.97
DU-145	102.30	104.36	106.07	104.95	105.82	104.37	108.56	109.06	109.49	107.49
**Breast Cancer**										
MCF7	94.08	75.65	76.01	52.33	63.50	18.43	64.15	72.14	77.50	78.24
MDA-MB-231/ATCC	90.37	67.79	70.05	69.17	-55.36	34.26	65.07	33.35	69.39	88.28
HS 578T	107.78	96.10	96.71	99.37	96.90	91.24	97.37	97.61	99.68	97.27
BT-549	99.14	106.19	104.50	110.26	100.83	96.44	100.28	112.25	107.41	100.66
T-47D	100.35	4.47	15.71	17.58	-33.87	-35.26	41.59	-8.45	30.30	80.52
MDA-MB-468	93.42	-43.13	-4.77	-61.36	-58.96	-65.71	1.26	-66.65	36.26	28.14

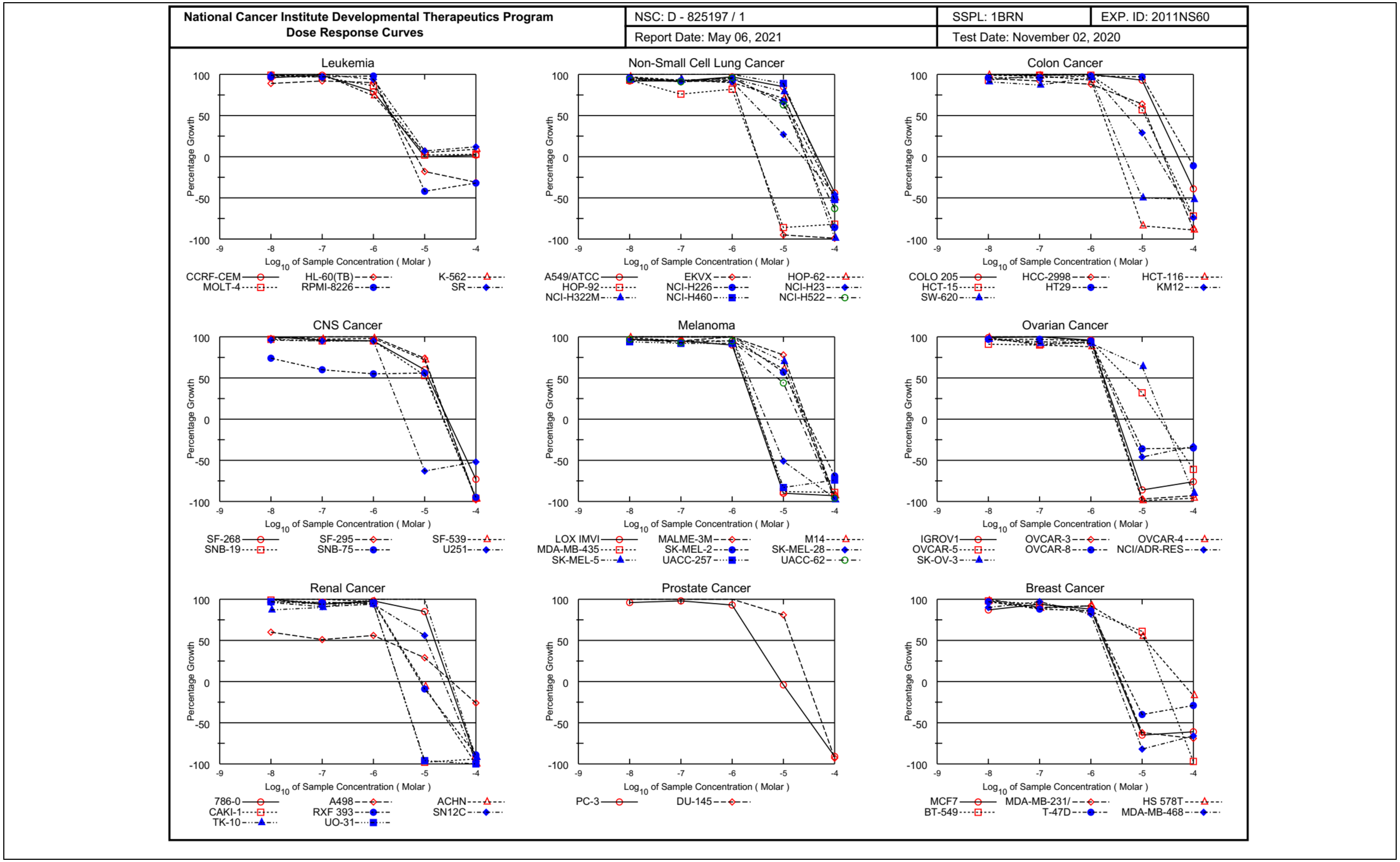

Figure S21: Dose response curves of five-dose *in vitro* antiproliferative activity of **BrPQ5**

National Cancer Institute Developmental Therapeutics Program
In-Vitro Testing Results

NSC : D - 825197 / 1	Experiment ID : 2011NS60	Test Type : 08	Units : Molar
Report Date : May 06, 2021	Test Date : November 02, 2020	QNS :	MC :
COMI : BMQ02	Stain Reagent : SRB Dual-Pass Related	SSPL : 1BRN	

	Log10 Concentration															
	Time		Mean Optical Densities					Percent Growth								
Panel/Cell Line	Zero	Ctrl	-8.0	-7.0	-6.0	-5.0	-4.0	-8.0	-7.0	-6.0	-5.0	-4.0	GI50		TGI	LC50
**Leukemia**																
CCRF-CEM	0.448	2.607	2.526	2.579	2.151	0.450	0.487	96	99	79	.	2	2.33E-6	>	1.00E-4	> 1.00E-4
HL-60(TB)	0.657	3.117	2.852	2.911	2.862	0.540	0.455	89	92	90	-18	-31	2.34E-6		6.82E-6	> 1.00E-4
K-562	0.127	1.628	1.598	1.637	1.244	0.207	0.262	98	101	74	5	9	2.25E-6	>	1.00E-4	> 1.00E-4
MOLT-4	0.482	2.985	2.960	2.902	2.623	0.540	0.566	99	97	86	2	3	2.67E-6	>	1.00E-4	> 1.00E-4
RPMI-8226	0.715	2.793	2.741	2.732	2.761	0.413	0.484	97	97	98	-42	-32	2.21E-6		5.01E-6	> 1.00E-4
SR	0.221	1.117	1.107	1.140	1.064	0.285	0.332	99	102	94	7	12	3.21E-6	>	1.00E-4	> 1.00E-4
**Non-Small Cell Lung Cancer**																
A549/ATCC	0.405	2.278	2.132	2.123	2.228	2.000	0.227	92	92	97	85	-44	1.87E-5		4.57E-5	> 1.00E-4
EKVX	0.833	2.330	2.270	2.196	2.220	0.044	0.011	96	91	93	-95	-99	1.69E-6		3.12E-6	5.77E-6
HOP-62	0.686	2.306	2.258	2.190	2.147	1.841	0.342	97	93	90	71	-50	1.50E-5		3.86E-5	9.97E-5
HOP-92	1.222	1.528	1.507	1.454	1.473	0.171	0.222	93	76	82	-86	-82	1.55E-6		3.08E-6	6.10E-6
NCI-H226	0.714	1.847	1.803	1.756	1.786	1.479	0.103	96	92	95	67	-86	1.30E-5		2.76E-5	5.85E-5
NCI-H23	0.583	2.277	2.176	2.157	2.125	1.046	0.318	94	93	91	27	-46	4.41E-6		2.37E-5	> 1.00E-4
NCI-H322M	0.802	2.369	2.267	2.264	2.289	2.039	0.009	93	93	95	79	-99	1.46E-5		2.78E-5	5.31E-5
NCI-H460	0.331	2.968	2.957	3.064	2.989	2.674	0.158	100	104	101	89	-52	1.88E-5		4.26E-5	9.61E-5
NCI-H522	1.044	2.868	2.768	2.708	2.794	2.196	0.391	94	91	96	63	-63	1.27E-5		3.18E-5	7.95E-5
**Colon Cancer**																
COLO 205	0.541	2.268	2.323	2.253	2.273	2.153	0.332	103	99	100	93	-39	2.13E-5		5.10E-5	> 1.00E-4
HCC-2998	0.804	2.971	2.873	2.798	2.702	2.201	0.099	95	92	88	64	-88	1.24E-5		2.65E-5	5.65E-5
HCT-116	0.243	2.452	2.431	2.416	2.302	0.040	0.027	99	98	93	-84	-89	1.76E-6		3.37E-6	6.46E-6
HCT-15	0.302	2.381	2.226	2.350	2.368	1.493	0.084	93	99	99	57	-72	1.14E-5		2.77E-5	6.74E-5
HT29	0.365	2.395	2.313	2.318	2.354	2.337	0.326	96	96	98	97	-11	2.74E-5		7.96E-5	> 1.00E-4
KM12	0.614	3.022	3.060	3.075	3.012	1.308	0.157	102	102	100	29	-74	5.02E-6		1.90E-5	5.80E-5
SW-620	0.266	2.205	2.027	1.961	2.135	0.133	0.129	91	87	96	-50	-52	2.07E-6		4.54E-6	9.97E-6
**CNS Cancer**																
SF-268	1.116	2.839	2.837	2.779	2.758	2.151	0.299	100	96	95	60	-73	1.19E-5		2.82E-5	6.70E-5
SF-295	0.392	2.242	2.201	2.303	2.362	1.767	0.009	98	103	106	74	-98	1.38E-5		2.70E-5	5.28E-5
SF-539	0.511	1.885	1.882	1.843	1.861	1.498	0.018	100	97	98	72	-97	1.35E-5		2.67E-5	5.29E-5
SNB-19	0.555	2.039	1.997	1.958	1.968	1.349	0.021	97	95	95	53	-96	1.05E-5		2.28E-5	4.91E-5
SNB-75	1.652	2.442	2.234	2.125	2.086	2.098	0.076	74	60	55	56	-95	1.10E-5		2.35E-5	5.02E-5
U251	0.378	2.026	1.956	1.944	1.945	0.139	0.181	96	95	95	-63	-52	1.93E-6		3.98E-6	8.24E-6
**Melanoma**																
LOX IMVI	0.281	2.082	2.034	1.984	1.910	0.028	0.021	97	95	90	-90	-93	1.67E-6		3.17E-6	5.99E-6
MALME-3M	0.783	1.859	1.816	1.801	1.857	1.620	0.044	96	95	100	78	-94	1.45E-5		2.83E-5	5.52E-5
M14	0.447	2.025	2.005	1.945	1.951	1.405	0.032	99	95	95	61	-93	1.17E-5		2.48E-5	5.26E-5
MDA-MB-435	0.585	2.658	2.678	2.689	2.738	0.072	0.062	101	102	104	-88	-89	1.91E-6		3.49E-6	6.36E-6
SK-MEL-2	1.367	3.045	3.044	3.066	3.037	2.323	0.423	100	101	100	57	-69	1.14E-5		2.83E-5	7.06E-5
SK-MEL-28	0.590	1.995	2.026	2.030	2.094	0.290	0.024	102	102	107	-51	-96	2.30E-6		4.76E-6	9.88E-6
SK-MEL-5	0.955	3.315	3.325	3.317	3.316	2.598	0.015	100	100	100	70	-98	1.31E-5		2.60E-5	5.15E-5
UACC-257	1.398	2.942	2.855	2.821	2.826	0.243	0.368	94	92	92	-83	-74	1.75E-6		3.37E-6	6.51E-6
UACC-62	0.867	2.912	2.843	2.774	2.811	1.774	0.031	97	93	95	44	-96	7.74E-6		2.07E-5	4.68E-5
**Ovarian Cancer**																
IGROV1	0.484	2.048	2.033	2.095	1.990	0.070	0.115	99	103	96	-86	-76	1.80E-6		3.39E-6	6.38E-6
OVCAR-3	0.568	2.030	2.112	2.038	1.959	0.020	0.040	106	101	95	-97	-93	1.72E-6		3.13E-6	5.72E-6
OVCAR-4	0.796	1.778	1.772	1.676	1.665	0.010	0.030	99	90	88	-99	-96	1.60E-6		2.97E-6	5.49E-6
OVCAR-5	0.430	1.483	1.384	1.375	1.421	0.762	0.166	91	90	94	32	-61	5.07E-6		2.18E-5	7.54E-5
OVCAR-8	0.507	2.379	2.324	2.325	2.295	0.324	0.328	97	97	95	-36	-35	2.22E-6		5.31E-6	> 1.00E-4
NCI/ADR-RES	0.407	1.722	1.677	1.649	1.630	0.219	0.274	97	94	93	-46	-33	2.04E-6		4.65E-6	> 1.00E-4
SK-OV-3	1.283	2.505	2.472	2.402	2.411	2.066	0.129	97	92	92	64	-90	1.23E-5		2.61E-5	5.50E-5
**Renal Cancer**																
786-0	0.632	2.458	2.451	2.349	2.429	2.191	0.038	100	94	98	85	-94	1.57E-5		2.99E-5	5.69E-5
A498	1.681	2.334	2.072	2.015	2.044	1.873	1.237	60	51	56	29	-26	1.62E-6		3.36E-5	> 1.00E-4
ACHN	0.403	1.871	1.898	1.906	1.831	0.379	-0.001	102	102	97	-6	-100	2.87E-6		8.76E-6	2.94E-5
CAKI-1	0.490	2.043	2.022	1.989	1.972	0.009	0.028	99	96	95	-98	-94	1.72E-6		3.11E-6	5.63E-6
RXF 393	0.694	1.490	1.467	1.462	1.455	0.629	0.075	97	96	96	-9	-89	2.71E-6		8.13E-6	3.23E-5
SN12C	0.457	1.752	1.696	1.632	1.681	1.185	0.012	96	91	94	56	-97	1.10E-5		2.32E-5	4.91E-5
TK-10	0.969	2.023	1.887	1.923	2.146	2.500	0.074	87	90	112	145	-92	2.52E-5		4.09E-5	6.63E-5
UO-31	0.655	2.391	2.336	2.286	2.309	0.029	-0.002	97	94	95	-96	-100	1.73E-6		3.16E-6	5.77E-6
**Prostate Cancer**																
PC-3	0.533	2.082	2.018	2.055	1.977	0.511	0.046	96	98	93	-4	-91	2.78E-6		9.05E-6	3.35E-5
DU-145	0.300	1.459	1.519	1.494	1.504	1.236	0.021	105	103	104	81	-93	1.50E-5		2.91E-5	5.65E-5
**Breast Cancer**																
MCF7	0.305	2.060	1.837	1.949	1.858	0.107	0.119	87	94	88	-65	-61	1.78E-6		3.77E-6	7.98E-6
MDA-MB-231/ATCC	0.563	1.469	1.487	1.381	1.398	0.213	0.174	102	90	92	-62	-69	1.88E-6		3.96E-6	8.34E-6
HS 578T	1.432	2.197	2.179	2.110	2.138	1.854	1.182	98	89	92	55	-17	1.18E-5		5.75E-5	> 1.00E-4
BT-549	1.107	2.309	2.281	2.242	2.132	1.844	0.032	98	94	85	61	-97	1.18E-5		2.44E-5	5.04E-5
T-47D	1.309	2.836	2.796	2.658	2.626	0.782	0.923	97	88	86	-40	-29	1.93E-6		4.81E-6	> 1.00E-4
MDA-MB-468	0.786	1.685	1.592	1.659	1.521	0.144	0.266	90	97	82	-82	-66	1.56E-6		3.16E-6	6.40E-6

Figure S22: Five-dose *in vitro* antiproliferative activity of **BrPQ5**

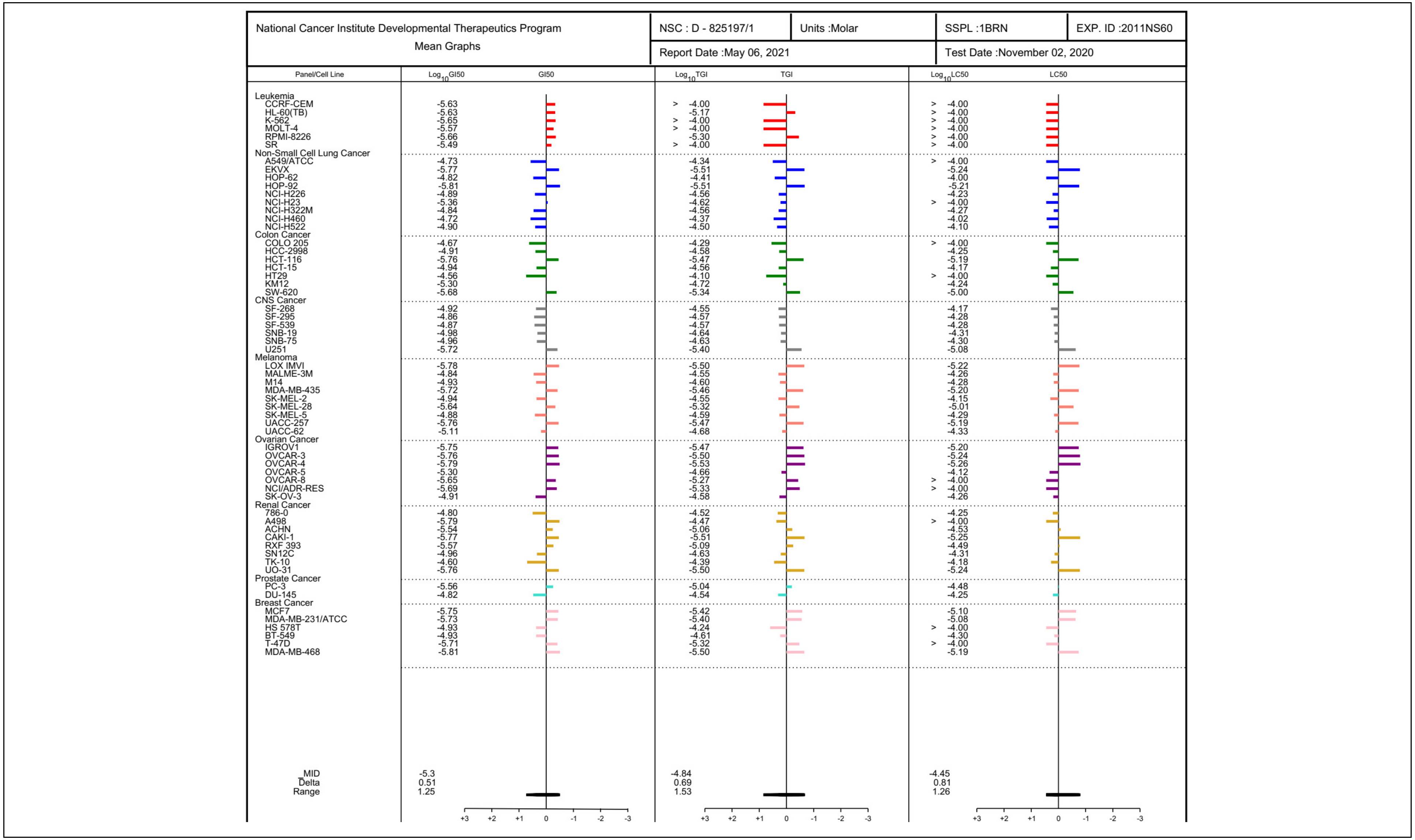

Figure S23: Data of five-dose *in vitro* antiproliferative activity of **BrPQ5**

The BrPQ analog (BrPQ5)

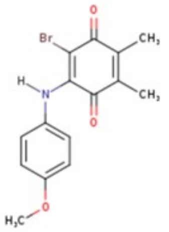

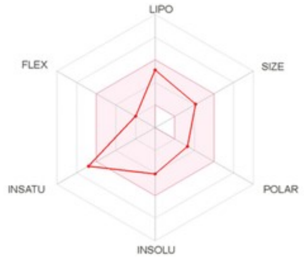

SMILES COc1ccc(cc1)NC1=C(Br)C(=O)C(=C(C1=O)C)C

Physicochemical Properties	
Formula	C15H14BrNO3
Molecular weight	336.18 g/mol
Num. heavy atoms	20
Num. arom. heavy atoms	6
Fraction Csp3	0.20
Num. rotatable bonds	3
Num. H-bond acceptors	3
Num. H-bond donors	1
Molar Refractivity	81.10
TPSA	55.40 Å²

Lipophilicity	
Log $P_{o/w}$ (iLOGP)	2.59
Log $P_{o/w}$ (XLOGP3)	3.40
Log $P_{o/w}$ (WLOGP)	3.01
Log $P_{o/w}$ (MLOGP)	1.37
Log $P_{o/w}$ (SILICOS-IT)	3.38
Consensus Log $P_{o/w}$	2.75

Water Solubility	
Log *S* (ESOL)	-4.09
Solubility	2.73e-02 mg/ml ; 8.12e-05 mol/l
Class	Moderately soluble
Log *S* (Ali)	-4.24
Solubility	1.92e-02 mg/ml ; 5.72e-05 mol/l
Class	Moderately soluble
Log *S* (SILICOS-IT)	-5.56
Solubility	9.30e-04 mg/ml ; 2.77e-06 mol/l
Class	Moderately soluble

Pharmacokinetics	
GI absorption	High
BBB permeant	Yes
P-gp substrate	No
CYP1A2 inhibitor	Yes
CYP2C19 inhibitor	Yes
CYP2C9 inhibitor	Yes
CYP2D6 inhibitor	No
CYP3A4 inhibitor	Yes
Log K_p (skin permeation)	-5.94 cm/s

Druglikeness	
Lipinski	Yes; 0 violation
Ghose	Yes
Veber	Yes
Egan	Yes
Muegge	Yes
Bioavailability Score	0.55

Medicinal Chemistry	
PAINS	2 alerts: ene_one_hal, quinone_A
Brenk	1 alert: chinone_1
Leadlikeness	Yes
Synthetic accessibility	3.09

Figure S24. The physicochemical properties and pharmacokinetic profile of the BrPQ analog (**BrPQ5**) evaluated using SwissADME

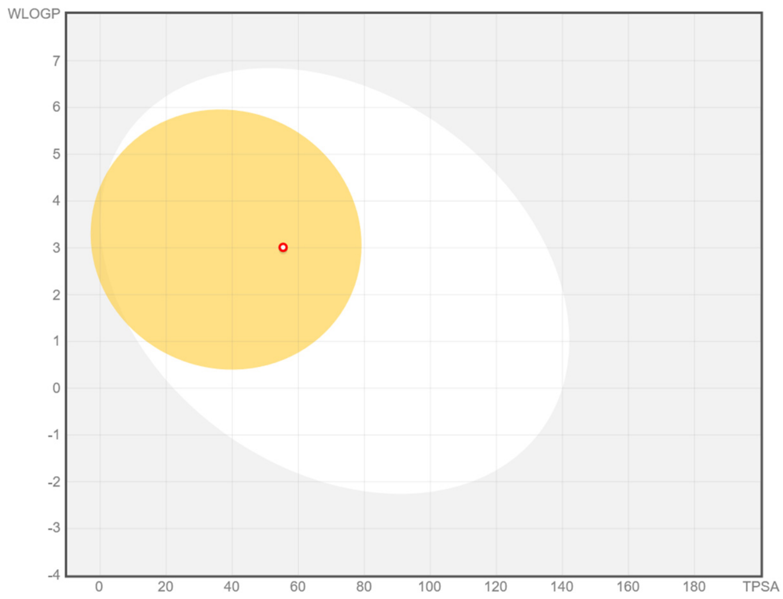

Figure S25. The generated BOILED-Egg graph of the BrPQ analog (**BrPQ5**) using SwissADME

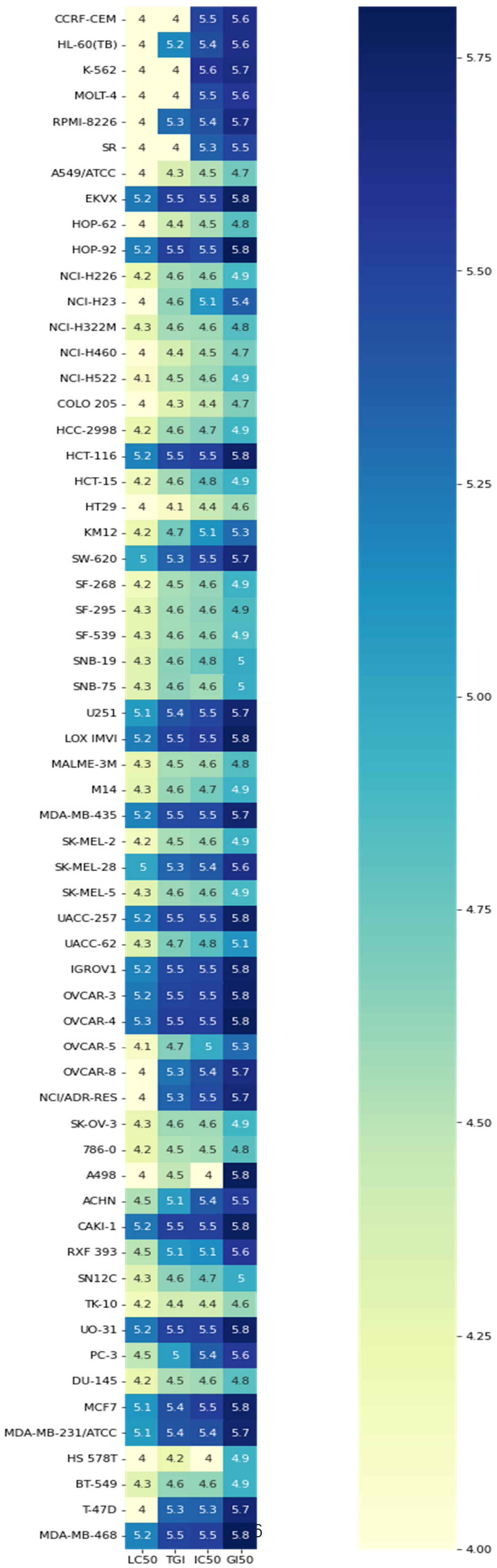
LC50 TGI IC50 GI50
CCRF-CEM 4 4 5.5 5.6
HL-60(TB) 4 5.2 5.4 5.6
K-562 4 4 5.6 5.7
MOLT-4 4 4 5.5 5.6
RPMI-8226 4 5.3 5.4 5.7
SR 4 4 5.3 5.5
A549/ATCC 4 4.3 4.5 4.7
EKVX 5.2 5.5 5.5 5.8
HOP-62 4 4.4 4.5 4.8
HOP-92 5.2 5.5 5.5 5.8
NCI-H226 4.2 4.6 4.6 4.9
NCI-H23 4 4.6 5.1 5.4
NCI-H322M 4.3 4.6 4.6 4.8
NCI-H460 4 4.4 4.5 4.7
NCI-H522 4.1 4.5 4.6 4.9
COLO 205 4 4.3 4.4 4.7
HCC-2998 4.2 4.6 4.7 4.9
HCT-116 5.2 5.5 5.5 5.8
HCT-15 4.2 4.6 4.8 4.9
HT29 4 4.1 4.4 4.6
KM12 4.2 4.7 5.1 5.3
SW-620 5 5.3 5.5 5.7
SF-268 4.2 4.5 4.6 4.9
SF-295 4.3 4.6 4.6 4.9
SF-539 4.3 4.6 4.6 4.9
SNB-19 4.3 4.6 4.8 5
SNB-75 4.3 4.6 4.6 5
U251 5.1 5.4 5.5 5.7
LOX IMVI 5.2 5.5 5.5 5.8
MALME-3M 4.3 4.5 4.6 4.8
M14 4.3 4.6 4.7 4.9
MDA-MB-435 5.2 5.5 5.5 5.7
SK-MEL-2 4.2 4.5 4.6 4.9
SK-MEL-28 5 5.3 5.4 5.6
SK-MEL-5 4.3 4.6 4.6 4.9
UACC-257 5.2 5.5 5.5 5.8
UACC-62 4.3 4.7 4.8 5.1
IGROV1 5.2 5.5 5.5 5.8
OVCAR-3 5.2 5.5 5.5 5.8
OVCAR-4 5.3 5.5 5.5 5.8
OVCAR-5 4.1 4.7 5 5.3
OVCAR-8 4 5.3 5.4 5.7
NCI/ADR-RES 4 5.3 5.5 5.7
SK-OV-3 4.3 4.6 4.6 4.9
786-0 4.2 4.5 4.5 4.8
A498 4 4.5 4 5.8
ACHN 4.5 5.1 5.4 5.5
CAKI-1 5.2 5.5 5.5 5.8
RXF 393 4.5 5.1 5.1 5.6
SN12C 4.3 4.6 4.7 5
TK-10 4.2 4.4 4.4 4.6
UO-31 5.2 5.5 5.5 5.8
PC-3 4.5 5 5.4 5.6
DU-145 4.2 4.5 4.6 4.8
MCF7 5.1 5.4 5.5 5.8
MDA-MB-231/ATCC 5.1 5.4 5.4 5.7
HS 578T 4 4.2 4 4.9
BT-549 4.3 4.6 4.6 4.9
T-47D 4 5.3 5.3 5.7
MDA-MB-468 5.2 5.5 5.5 5.8
5.75
5.50
5.25
5.00
4.75
4.50
4.25
4.00

Figure S26. The heatmap illustrating the five-dose *in vitro* antiproliferative activity of **BrPQ5**
